# Structural and biochemical analysis of the PARP1-homology region of PARP4/vault PARP

**DOI:** 10.1093/nar/gkad1064

**Published:** 2023-11-16

**Authors:** Léonie Frigon, John M Pascal

**Affiliations:** Department of Biochemistry and Molecular Medicine, Université de Montréal, Montréal, Qc H3T 1J4, Canada; Department of Biochemistry and Molecular Medicine, Université de Montréal, Montréal, Qc H3T 1J4, Canada

## Abstract

PARP4 is an ADP-ribosyltransferase that resides within the vault ribonucleoprotein organelle. Our knowledge of PARP4 structure and biochemistry is limited relative to other PARPs. PARP4 shares a region of homology with PARP1, an ADP-ribosyltransferase that produces poly(ADP-ribose) from NAD^+^ in response to binding DNA breaks. The PARP1-homology region of PARP4 includes a BRCT fold, a WGR domain, and the catalytic (CAT) domain. Here, we have determined X-ray structures of the PARP4 catalytic domain and performed biochemical analysis that together indicate an active site that is open to NAD^+^ interaction, in contrast to the closed conformation of the PARP1 catalytic domain that blocks access to substrate NAD^+^. We have also determined crystal structures of the minimal ADP-ribosyltransferase fold of PARP4 that illustrate active site alterations that restrict PARP4 to mono(ADP-ribose) rather than poly(ADP-ribose) modifications. We demonstrate that PARP4 interacts with vault RNA, and that the BRCT is primarily responsible for the interaction. However, the interaction does not lead to stimulation of mono(ADP-ribosylation) activity. The BRCT–WGR–CAT of PARP4 has lower activity than the CAT alone, suggesting that the BRCT and WGR domains regulate catalytic output. Our study provides first insights into PARP4 structure and regulation and expands understanding of PARP structural biochemistry.

## Graphical Abstract

**Graphical Abstract:**
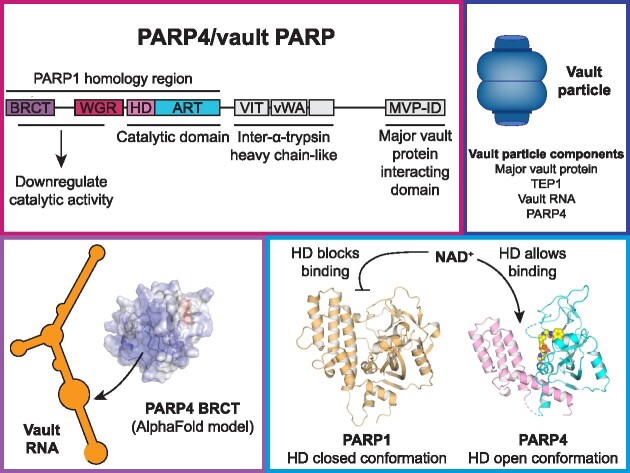

## Introduction

The cytoplasm of many eukaryotic cell types contains a ribonucleoprotein organelle known as vault (1,2). Vault is noteworthy for its gigantic size (∼13 MDa) and its hollow, barrel-like structure (3,4). There is still no clear indication of vault function in normal and pathological contexts, although studies have suggested potential functions in transport, signal transmission, cellular stress protection, immune response and chemotherapy resistance (5,6). Vault is primarily composed of major vault protein. A 3.5 Å X-ray structure of vault demonstrated that 78 molecules of major vault protein assemble into two equivalent half particles, each half containing 39 molecules and exhibiting 39-fold symmetry (7). Vault particles contain ∼100-nucleotide RNA molecules known as vault RNA (vtRNA) of which there are four known varieties (vtRNA1-1, vtRNA1-2, vtRNA1-3 and vtRNA2-1) (8). Besides major vault protein, telomerase-associated protein (TEP1) and PARP4 (or vault PARP) are the other protein components of vault. Each vault is estimated to contain two copies of TEP1, several copies of vtRNA and eight copies of PARP4 (9).

PARP4 is an ADP-ribosyltransferase that uses NAD^+^ as a substrate to synthesize ADP-ribose modifications on molecules (10,11). The term PARP historically stood for poly(ADP-ribose) polymerase, but the term now refers to the structurally-related, mammalian ADP-ribosyltransferases that catalyze either mono(ADP-ribosylation) (MARylation) or poly(ADP-ribosylation) (PARylation) (10,11). Indeed, most of the canonical 17 human PARP enzymes catalyze MARylation, and PARylation is catalyzed by only a subset: PARP1, PARP2, TNKS1 (PARP5A) and TNKS2 (PARP5B) (12). A defining structural feature of the PARP ADP-ribosyltransferase group is a catalytic triad motif that participates in binding NAD^+^ and partly determines the capacity to perform MARylation versus PARylation. The triad H-Y-E is generally indicative of PARylation, and MARylation is associated with the triad H-Y-[I/L/Y]. However, PARP3 and PARP4 contain the catalytic triad H-Y-E but are considered MARylation enzymes (12,13), indicating that other structural features are important determinants of catalytic output.

**Figure 1.**
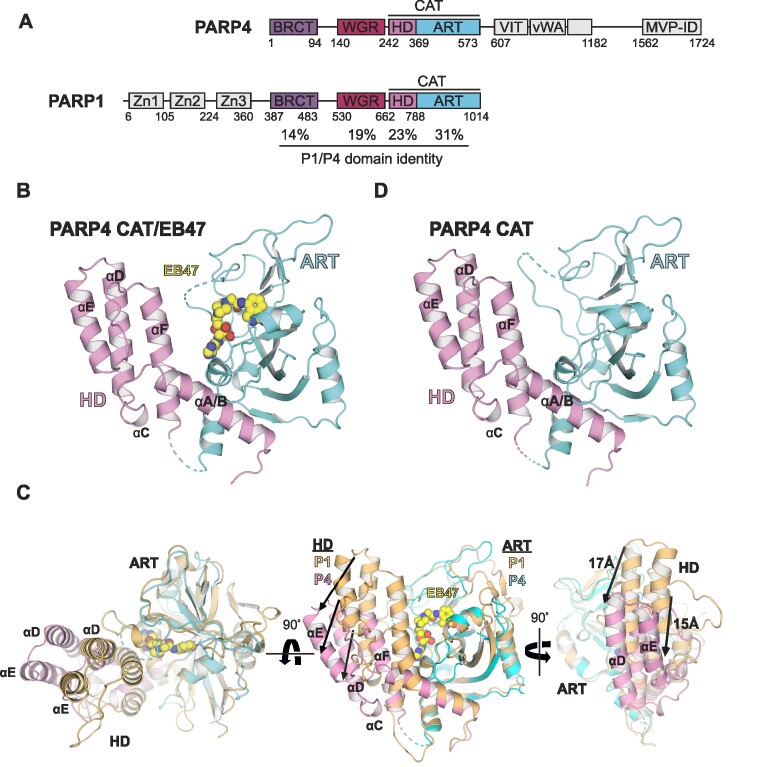
Crystal structure of the PARP4 catalytic domain and comparison to PARP1. (**A**) Schematic representation of human PARP4 and human PARP1 domain compositions. Approximate residue numbers at domain boundaries are shown below each protein. The percentage of identity between PARP1 and PARP4 in the BRCT, WGR, HD and CAT domains are shown. See text for domain definitions. (**B**) Crystal structure of the PARP4 catalytic domain bound to EB47 at 2.95 Å resolution (PDB: 8SX2; from this study). The HD is depicted in pink and the ART domain in cyan. EB47 is shown as spheres with yellow carbons. The continuous αA/B helix is labeled. (**C**) Comparison of the catalytic domain of PARP1 (drawn in light orange, PDB: 7AAA) (22) and the catalytic domain of PARP4 bound to EB47 (HD drawn in pink, ART drawn in cyan, EB47 in yellow). The two structures were aligned by the ART domain, highlighting the translation and rotation of the PARP4 HD relative to the HD of PARP1. Three views of the aligned structures are shown. The arrows connect PARP1 residues to the equivalent residues of PARP4. Lengths are indicated for two of the arrows in the rightmost panel. (**D**) Crystal structure of the PARP4 catalytic domain without ligand at 4.2 Å resolution (PDB: 8SX1; from this study). The HD is depicted in pink and the ART domain in cyan.

PARP4 belongs to a distinct clade among the mammalian PARPs (14). This distinction is largely due to the C-terminal two-thirds of the protein that contains a vault inter-alpha-trypsin domain (VIT), a Von Willebrand factor type A domain (vWA) and a major vault protein interaction domain (MVP-ID) (Figure 1A). The MVP-ID allows PARP4 to localize to the inside of the vault organelles (3,15). VIT and vWA are conserved domains in inter-alpha-trypsin inhibitor heavy chains (ITIH) (16), but their function in PARP4 are unknown. Based on this PARP4 homology to ITIH proteins, the name ITIH-like (ITIHL) region has been proposed for this central part of PARP4 (17). The N-terminal one-third of PARP4 bears resemblance to other PARP family members, in particular sharing a region of homology with PARP1 (Figure 1A). PARP1 is a multi-function protein, with a major impact on the cellular response to DNA damage. PARP1 detects DNA strand break damage and the interaction with breaks potently stimulates PARylation activity. The catalytic ADP-ribosyltransferase (ART) fold of PARP1 is regulated by a helical domain (HD), a WGR domain (named for conserved residues) and three zinc-finger domains (Zn1, Zn2, Zn3). The zinc fingers and WGR are organized on DNA strand breaks and engage the HD in a manner that distorts specific HD helices, thereby reversing an autoinhibitory conformation of the HD that blocks NAD^+^ substrate access to the ART fold (18–22). Sequence analysis and AlphaFold structure prediction indicate that PARP4 contains both HD and WGR domains, as noted in a recent study analyzing PARP enzyme domain using AlphaFold (17). The WGR-HD-ART domain combination is also found in PARP2 and PARP3 that are involved in the DNA damage response. PARP1 also contains a BRCT fold that is not involved in the activation mechanism in response to DNA breaks, but does bind to intact duplex DNA (23). PARP4 is also expected to contain a BRCT fold based on sequence analysis and structure prediction (17). Thus, PARP4 and PARP1 share a region of homology: the BRCT–WGR–CAT collection of domains.

We undertook an analysis of the PARP1-homology region of PARP4 to explore the potential similarities and differences in biochemistry and structure. Here, we have determined crystal structures of the PARP4 catalytic region that indicate that PARP4 indeed contains an HD domain, but the HD conformation is quite distinct from PARP1 and biochemical analysis indicates a more ‘open’ PARP4 ART active site relative to PARP1. We have also determined crystal structures of the PARP4 ART fold that illustrate that multiple alterations to the ‘acceptor’ site have rendered PARP4 incapable of PARylation, despite the H-Y-E motif. In examining the capacity of PARP4 to bind to nucleic acids, in analogy to PARP1, we found that the BRCT domain of PARP4 interacts with nucleic acid, in particular showing a preference for vtRNA. The interaction with vtRNA does not modulate the MARylation activity of PARP4, in stark contrast to the potent activation of PARP1 in response to interaction with DNA strand breaks. However, the BRCT and WGR domains do appear to modulate the catalytic output of PARP4, with BRCT–WGR–CAT exhibiting lower activity than the CAT domain alone. Collectively, the study provides the first structural views of PARP4, an informative biochemical analysis of the PARP1-homology region of PARP4 and new insights into PARP family regulation.

## Materials and methods

### Expression constructs and mutagenesis

Human PARP4 residues 242–573 (i.e. catalytic domain, CAT) were expressed from a pET28 vector with an N-terminal his-tag and SMT sumo-like tag (custom gene synthesis by BioBasic). Site-directed mutagenesis was performed using the QuikChange protocol (Agilent) and verified by automated Sanger sequencing. PARP4 CATΔHD was prepared by deleting the HD (residues 282–364) and adding an 8-residue linker (GSGSGSGG). A pET28 expression vector was used to produce the BRCT–WGR–CAT fragments of PARP4 with an N-terminal his-tag and SUMO-tag (gift from Dr Michael Cohen). The BRCT domain was deleted from this construct using QuikChange mutagenesis to yield the WGR–CAT construct. The N-terminal helix (residues 242–255) was deleted from the CAT and CATΔHD constructs using QuickChange mutagenesis to yield the CATΔN-term and CATΔHDΔN-term constructs.

### Protein expression and purification

PARP4 CAT, PARP4 CATΔHD, PARP4 CATΔN-term and PARP4 CATΔHDΔN-term were expressed in *Escherichia coli* strain Rosetta2(DE3) (Novagen). The cells were grown at 37°C in LB medium until the OD_600_ reached ∼0.6, and then induced with 200 μm isopropyl β-d-thiogalactopyranoside at 16°C for 20 h. The cells were resuspended in 20 mM HEPES, pH 8.0, 500 mM NaCl, 0.5 mm Tris[2-carboxyethyl] phosphine (TCEP), 0.1% Nonidet *P*-40 (NP40), 10% glycerol, 1 mm phenylmethylsulfonyl fluoride (PMSF), protease inhibitors (antipain, pepstatine, leupetine, benzamidine and aprotinine), and then lysed using a cell disrupter (Avestin). The lysate was then centrifugated for 2 h at 40 000 × g. The supernatant was filtered then loaded onto a 5-ml HP chelating column (Cytiva) charged with Ni(II) and pre-equilibrated with lysis buffer containing 10% glycerol. The column was washed with 10 column volumes of 20 mM HEPES, pH 8.0, 500 mM NaCl, 0.5 mM TCEP, 1 mM PMSF, protease inhibitors, 10% glycerol and 20 mM imidazole. The column was then washed with 10 column volumes of the same buffer supplemented with 1 M NaCl, and then again with 10 column volumes of 500 mM NaCl buffer. The proteins were then eluted with 4 column volumes of buffer supplemented with 400 mM imidazole. The N-terminal tag was cut using ULP1 protease during dialysis into 20 mM HEPES pH 8.0, 150 mM NaCl, 0.5 mM TCEP and 10% glycerol. The protein mixture was then passed over a second Ni(II)-affinity column, with desired protein in the flow-through and the tag remaining on the column. The flow-through protein was then passed over a size-exclusion chromatography column mounted on a chromatography system (AKTA purifier) and maintained in 20 mM HEPES pH 8.0, 150 mM NaCl, 0.1 mM TCEP and 0.1 mM EDTA. A Hiprep 26/60 Sephacryl S-200 column (Cytiva) was used for gel filtration of PARP4 CAT and PARP4 CATΔHD. A HiLoad 16/600 Superdex75 column (Cytiva) was used for PARP4 CATΔN-term and PARP4 CATΔHDΔN-term.

PARP4 BRCT–WGR–CAT and PARP4 WGR–CAT were expressed and purified as described above, with the exception that the N-terminal tag was cut using TEV protease and an additional chromatographic step was performed after Ni(II) affinity and prior to gel filtration. The dialyzed, protease-treated sample was diluted to a NaCl concentration of 75 mM through the slow addition of 25mM Tris pH 7.9, 0.1mM TCEP, 10% glycerol, 1 mM EDTA. A 5 ml HiTrap Q HP anion exchange column (Cytiva) was equilibrated with buffer A (75 mM NaCl, 25 mM Tris pH7.9, 0.1 mM TCEP, 10% glycerol and 1 mM EDTA). The diluted sample was then applied to the Q column. The Q column was placed on a chromatography system (AKTA) to elute the protein with the following three-step program: (i) 5 column volumes of buffer A, (ii) 40-column volume gradient from 0 to 100% of buffer B (1 M NaCl, 25 mM Tris pH7.9, 0.1 mM TCEP, 10% glycerol, 1 mM EDTA), and (iii) 5 column volumes of 100% buffer B. The fractions containing the protein were then pooled, concentrated and passed over a Hiprep 26/60 Sephacryl S200 column (Cytiva). PARP1 proteins were expressed and purified as described (24). Aliquots of concentrated proteins were flash-frozen in liquid nitrogen and stored at -80°C.

### Crystallization and structure determination

All diffraction data was processed using XDS (25) and AIMLESS (26) as implemented in the program package CCP4i (27) (Table 1). Model building was performed using COOT (28), and PHENIX (29) was used for refinement. X-ray diffraction data for PARP4 CAT domain in complex with EB47, as well as PARP4 CAT ΔHD bound to EB47 or NADH, was collected at SIBYLS beamline 8.3.1 (Advance Light Source). X-ray diffraction data for PARP4 CAT domain alone was collected on the Canadian Macromolecular Crystallography Facility bending magnet (CMCF-BM) of the Canadian Light Source.

**Table 1.** Crystallographic statistics

Data collection^a^				
Compound	CAT	CAT + EB47	CATΔHD + EB47	CATΔHD + NADH
PDB ID	8SX1	8SX2	8SWZ	8SWY
Space group	*P*12_1_1	*P*2_1_2_1_2_1_	*C*222_1_	*C*222_1_
Unit cell dimensions	*a*= 126.6 Å, *b*= 71.6 Å, *c*= 164.0 Å α=γ=90°, β=103.3°	*a*= 61.9 Å, *b*= 72.3 Å, *c*= 161.0 Å α=β=γ=90°	*a*= 116.8 Å, *b*= 150.9 Å, *c*= 104.6 Å α=β=γ=90°	*a*= 116.9 Å, *b*= 151.7 Å, *c*= 104.6 Å α=β=γ=90°
Wavelength (Å)	1.18	1.12	1.12	1.12
Resolution range (Å)	47.0–4.2 (4.54–4.2)	165.9–2.95 (3.13–2.95)	69.2–3.0 (3.2–3.0)	46.4–2.55 (2.66–2.55)
Completeness (%)	99.8 (99.9)	100.0 (100.0)	100 (99.9)	100 (100)
Unique observations	21 286 (4357)	15 882 (2510)	18 894 (3020)	30 692 (3729)
Average redundancy	4.6 (4.6)	14.2 (14.6)	14.5 (14.5)	9.0 (9.4)
Mean (*I*/σ*I*)^b^	6.1 (1.8)	11.6 (1.4)	18.2 (1.4)	19.6 (1.6)
*R*_merge_ (%)^b^	16.2 (78.9)	20.3 (223.5)	11.4 (202.2)	6.3 (142.6)
*R*_pim_ (%)^b^	8.4 (40.6)	5.6 (60.0)	31 (54.7)	22 (48.6)
Mean I CC(1/2)^b^	0.997 (0.687)	0.998 (0.541)	0.999 (0.521)	0.999 (0.612)
				
Model refinement^a^				
Resolution range (Å)	47.07–4.2 (4.3–4.2)	19.99–2.95 (3.04–2.95)	69.24–3.00 (3.08–3.00)	46.41–2.55 (2.59–2.55)
Number of reflections	21 241 (2884)	15 761 (2668)	18 874 (2808)	30 668 (2817)
*R* ^c^	0.2773 (0.3529)	0.2153 (0.3458)	0.2034 (0.3356)	0.2108 (0.4018)
*R*_free_^c^	0.3312 (0.4410)	0.2616 (0.3720)	0.2483 (0.4233)	0.2536 (0.4316)
Number of atoms/average *B*-factor (Å^2^)				
protein	19 488/187.17	4 861/96.32	5 663/111.91	5 678/97.93
solvent	3/59.94	37/68.47	47/94.14	75/94.77
inhibitor	-	78/69.05	78/90.67	88/84.36
Phi/Psi, preferred (%)/outliers (#)^d^	93.64/0	93.59/2	92.89/0	94.61/2
Rmsd bond angles (°)	1.257	1.099	1.559	1.299
Rmsd bond lengths (Å)	0.007	0.006	0.011	0.009

^a^Values in parentheses refer to data in the highest resolution shell.

^b^As calculated in SCALA (48): *R*_merge_ = ∑_*hkl*_∑_*j*_|*I*_*j*_ – 〈*I*〉| / ∑_*hkl*_∑_*j*_*I_j_*. 〈*I*〉 is the mean intensity of *j* observations of reflection *hkl* and its symmetry equivalents; *R*_pim_ takes into account measurement redundancy when calculating *R*_merge_; mean *I* CC(1/2) is the correlation between mean intensities calculated for two randomly chosen half-sets of the data.

^c^R = ∑_hkl_|F_obs_ – *k*F_calc_|/ ∑_hkl_|F_obs_| for reflections used in refinement. R_free_ = R for 5% of reflections excluded from crystallographic refinement.

^d^As reported in COOT (28).

PARP4 CAT (10 mg/ml) in complex with EB47 was crystallized in 23% PEG3350, 0.1 M Bis–Tris pH 6.5, 0.2 M MgCl_2_ and 600 μM EB47 using sitting drop vapor diffusion trays at room temperature. A cryo-protection solution (23% PEG3350, 0.1 M Bis–Tris pH 6.5, 0.2 M MgCl_2_, 600 μM EB47 and 30% glycerol) was exchanged with the crystallization solution before flash-cooling with liquid nitrogen. EB47 was purchased from Cayman Chemical (Cat. # 34684). The structure was determined by molecular replacement in PHASER-MR (30) from the PHENIX suite (31) using the PARP1 ART derived from PDB 4DQY as a search model.

PARP4 CAT (10 mg/ml) was crystallized in 23% PEG3350 and 0.1 M Bis–Tris pH 6.5 in sitting drop vapor diffusion trays at room temperature. The drop solution was exchange with a cryo-protection solution (23% PEG3350, 0.1 M Bis–Tris pH 6.5 and 15% glycerol) before the crystal was flash-cooled with liquid nitrogen. The structure was determined by molecular replacement in PHASER-MR (30) from the PHENIX suite (31) using a modified version of PARP4 CAT/EB47 as a search model.

PARP4 CATΔHD (40 mg/ml) bound to EB47 was crystallized in 20% PEG3350, 0.2 M sodium citrate tribasic dihydrate and 800 μM EB47 in sitting drop vapor diffusion trays at room temperature. A cryo-protection solution (20% PEG3350, 0.2 M sodium citrate tribasic dihydrate, 800 μM EB47 and 20% glycerol) was exchanged with the crystallization solution before flash-cooling with liquid nitrogen. The structure was determined by molecular replacement using PHASER-MR (30) from the PHENIX suite (31), using a modified version of the PARP4 CAT with EB47 structure as a search model.

PARP4 CATΔHD (40 mg/ml) bound to NADH was crystallized in 21% PEG3350, 0.2 M sodium citrate tribasic dihydrate, 1% ethylene glycol and 800 μM NADH in sitting drop vapor diffusion trays at room temperature. A cryo-protection solution (21% PEG3350, 0.2 M sodium citrate tribasic dihydrate, 1% ethylene glycol, 800 μM NADH and 15% glycerol) was exchanged with the crystallization solution before flash-cooling with liquid nitrogen. NADH was purchased from Bio Basic (Cat. # NB0642). The structure was determined by molecular replacement using PHASER-MR (30) from the PHENIX suite (31), using a modified version of the PARP4 CATΔHD with EB47 structure as a search model.

### Differential scanning fluorimetry (DSF)

Differential scanning fluorimetry experiments were performed using 5 μM protein and 5× Sypro Orange (Invitrogen) in a final volume of 20 μl. Fluorescence emission was measured while the temperature was increased from 20 to 85°C (0.02°C/s with 25 acquisitions per degree) in a Roche LightCycler 480 RT-PCR. Experiments were performed in the following buffer: 25 mM HEPES pH 8.0, 150 mM NaCl, 1 mM EDTA and 0.1 mM TCEP. A Boltzmann sigmoid was fit to the data to determine the apparent melting temperature (*T*_M_) values using MATLAB (MathWorks). Experiments were repeated three times. The non-hydrolyzable NAD^+^ analog benzamide adenine dinucleotide (BAD) was chemically synthesized by Viva Biotech Lmt. (Shanghai). Proton NMR spectra and liquid chromatography mass spectrometry confirmed the structure of the compound and the X-ray structure has been determined in complex with PARP1 (21).

### Nucleic acids

Vault RNA (vtRNA1-2: NCBI accession number NR_026704) was chemically synthesized by IDTDNA bearing a 5′-6-FAM fluorescent group. The vtRNA was annealed at 87 μm in a buffer consisting of 5 mM MES pH 6.5, 25 mM NaCl and 5 mM MgCl_2_ (annealing buffer). The 47-nucleotide DNAs were purchased from IDTDNA with the following complementary sequences:

5′-CAGATCTACCGAATCAGTCCGACGACGCATCTGCACTACGAGGATAC-3′,

5′-GTATCCTCGTAGTGCAGATGCGTCGTCGGACTGATTCGGTAGATCTG-3′.

For experiments using fluorescence detection, the first strand was synthesized with a Cy3 fluorescent label on the 5′ terminus. The 47-bp DNA duplex at 475 μm was formed by annealing the two strands in annealing buffer. The 47-nucleotide single-stand DNA with Cy3 label was annealed at 950 μm. A synthetic Hepatitis Delta Virus (HDV) ribozyme was a kind gift from the laboratory of Dr. Pascale Legault (Université de Montréal). The HDV RNA (84 ribonucleotides) was annealed at 68 μm. HDV RNA has the following sequence:

5′-GCCGGCCAUGGUCCCAGCCUCCUCGCUGGCGGCCGGUGGGCAACAUUCCGAGGG

GACCGUCCCUCGGUAAUGGCGAAUGGGACA-3′. The annealing procedure consisted of heating the samples to 95°C and then slowing lowering the temperature to 20°C over a period of one hour in a thermocycler.

### Western blot assay of mono(ADP-ribosylation) activity

For experiments without nucleic acids, PARP4 (2 μM) and/or PARP1 (2 μM) were mixed with NAD^+^ (100 μM or 5 mM) in a buffer consisting of 20 mM Tris pH 7.5, 50 mM NaCl, 5 mM MgCl_2_ and 0.1 mM TCEP. For experiments with nucleic acid, PARP4 (6 μM) was incubated with vtRNA (20 μm) for 30 min at room temperature, and the reactions were started by the addition of 100 μm NAD^+^ and then quenched at various time points with the addition of SDS-PAGE loading buffer (1X final). Quenched reactions were incubated for 5 min at 100°C and then resolved on 15% SDS-PAGE. The gel was transferred onto a nitrocellulose membrane that was then stained with Ponceau S and imaged for PARP4 protein content evaluation. The membrane was then blocked for 1 hour in Tris-buffered saline with Tween (TBS-T; 20 mM Tris pH 7.5, 150 mM NaCl and 0.1% Tween-20) supplemented with 5% evaporated cow's milk at room temperature. Blots were incubated for one hour at room temperature with a pan ADP-ribose binding reagent (MABE1016, Millipore Sigma, 1:1500) in 5% milk in TBS-T. Blots were washed three times in TBS-T for 5 minutes at room temperature. Next, blots were incubated with a secondary antibody (donkey anti-rabbit conjugated to HRP, Santa Cruz, sc2313, 1:7000) in 1% milk in TBS-T for 1 h at room temperature. Blots were washed three times in TBS-T for 5 min at room temperature. The signal was revealed using enhanced chemiluminescence (ECL; Bio-Rad). The band intensities were measured and normalized to the amount of PARP4 protein based on Ponceau S staining intensities. The reported data represents the average and standard deviations of the normalized intensities from three repeats of each experiment. Evaluation of a dilution series of a reaction condition yielding an intense signal indicated that all the reaction measurements lie close to the linear response range for this assay (Figure S1) thus supporting the relative comparison of PARP4 reactions.

### Fluorescence polarization (FP) binding assay

For measuring PARP4 affinity for vtRNA, increasing concentrations of PARP4 BRCT–WGR–CAT or WGR–CAT were incubated for 30 minutes at room temperature with 30 nM vtRNA. The fluorescence polarization assay was performed in 50 mM Tris pH7.5, 0.05% NP40, 2 mM DTT, 50 mM NaCl and 5% glycerol. Fluorescence polarization was measured on a VictorV plate reader (Perkin Elmer). A 1:1 binding model was fit to the data in MATLAB (MathWorks). Three independent experiments were performed for each binding curve.

### Electrophoretic mobility shift assay (EMSA)

PARP4 (0–20 μm) was prepared on ice in 2X binding buffer (100 mM Tris pH 7.5, 100 mM NaCl and 40% glycerol). Annealed vtRNA, HDV RNA, 47-bp DNA duplex and 47-nucleotide single-stand DNA (200 nm) were prepared on ice in 2× RNA buffer (0.1% NP40 and 4 mM DTT). The samples were prepared by mixing equal parts of PARP4 and nucleic acid, yielding final concentrations of 100 nm nucleic acid, 50 mM Tris pH 7.5, 50 mM NaCl, 20% glycerol, 0.05% NP40, 2 mM DTT and PARP4 between 0 and 20 μm. The samples were incubated at room temperature for 30 min. Mini-PROTEAN TGX Stain-Free gels (Bio-RAD; 7.5%) were pre-run in Tris–glycine buffer at 4°C (100 V, 2 h), and then the loaded samples were resolved at 100 V for 2 h at 4°C. The nucleic acids were visualized by staining with the fluorescent dye SYBR Gold 1× in Tris–glycine buffer for 30 min, or using the fluorescent dye attached to the nucleic acid (as designated in figure legends). The fluorescent intensity of imaged gel bands was quantified in ImageJ (NIH). The percent bound was calculated as the ratio of shifted band intensity over the total band intensity measured (unshifted probe plus shifted band) multiplied by 100. A 1:1 binding model was fit to the data in MATLAB (MathWorks). Three independent experiments were performed for each binding curve.

## Results

### Crystal structures of the PARP4 catalytic domain

Our structural analysis first focused on the region of PARP4 bearing the ART domain and the expected HD (i.e. catalytic domain or CAT). We expressed and purified PARP4 residues 242–573 with an N-terminal SMT tag that was proteolytically removed during purification. The PARP4 catalytic domain was crystallized on its own, and in complex with the NAD^+^-mimic EB47 (32). The ART domain of PARP1 (derived from PDB code 4DQY) was used for molecular replacement phasing of the diffraction data from the CAT/EB47 crystals. The initial difference electron density maps were used to build the HD structure and to adjust the starting ART model.

The CAT/EB47 complex crystals diffracted to 2.95 Å and belong to space group *P*2_1_2_1_2_1_ with two molecules in the asymmetric unit (Table 1). The refined structure has a crystallographic *R*/*R*_FREE_ of 0.215/0.262 (Figure 1B). While building the model for the HD using the CAT/EB47 diffraction data, it was clear that the HD occupied a significantly different position relative to the ART than that observed with PARP1/2/3. This difference in relative orientation can be appreciated when the ART domain of PARP4 is aligned to the PARP1 ART from a CAT structure that represents the closed conformation (Figure 1C). In this closed conformation, the HD of PARP1 is held near the ART active site, in a position to block the binding of substrate NAD^+^. This is the structure adopted by PARP1 when it is not bound to DNA damage. In contrast to PARP1, the PARP4 HD is translated and rotated away from the ART active site, located in a position that does not appear to be an impediment to NAD^+^ binding (Figure 1C). We reasoned that the bound EB47 molecule could potentially influence the positioning of the HD relative to the ART, thus we sought a PARP4 CAT structure with no compound in the active site. Crystals of the PARP4 catalytic domain alone diffracted to 4.2 Å and belonged to space group *P*2_1_2_1_2_1_ with eight molecules in the asymmetric unit (Table 1). The refined structure has a crystallographic *R*/*R*_FREE_ of 0.277/0.331 (Figure 1D). Despite the modest resolution, the structure clearly indicated that PARP4 HD adopts a similar orientation relative to the ART regardless of active site occupancy. The HD conformation was consistently open in all copies of the CAT domain within the two crystal types (10 copies total). Since these copies occupy different crystal environments, we deem it unlikely that the open HD conformation is a product of crystallization. We undertook biochemical analysis of PARP4 to further explore the potential consequences of an open conformation of the HD in PARP4.

### The PARP4 HD does not fully auto-inhibit PARP4 catalytic activity

**Figure 2.**
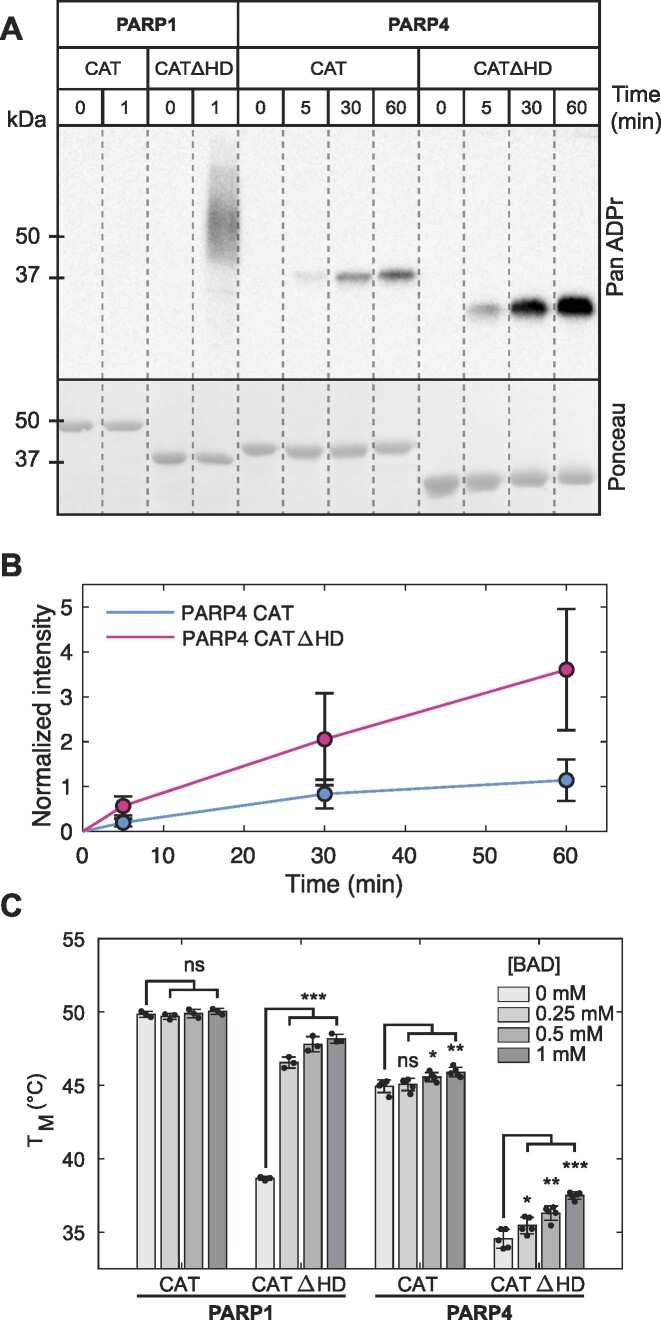
PARP4 catalytic domain adopts an open conformation that permits NAD^+^ binding. (**A**) Western blot catalytic activity assay measuring PARP1/PARP4 automodification. Purified PARP1 or PARP4 (2 μM) were incubated in the presence of 5 mM NAD^+^ for the indicated time points. A pan-ADP-ribose binding reagent was used for detection of modified proteins. Ponceau staining revealed the amount of protein in each reaction. A representative experiment is shown. The experiment was performed three times. (see also Supplementary Figure S2 for the reaction performed at 100 μM NAD^+^). (**B**) Quantification of the three repeats of the experiment shown in panel A. The PanADPr intensity was normalized to the Ponceau intensity measurements. The averages and standard deviations are plotted. (**C**) Differential scanning fluorimetry analysis. The experiments were performed with 5 μM PARP1 or PARP4 in the absence or presence of the indicated concentrations of BAD. *T*_M_ values represent the apparent melting temperature of the protein in the presence or absence of BAD. The individual measurements from repeat experiments are plotted. The bars represent the averages and error bars represent standard deviations. Two-sample, two-sided *t*-tests were used to compare the *T*_M_ values. * *P* < 0.05, ** *P* < 0.005, *** *P* < 0.0005, and ns indicates not significant.

The structures of the PARP4 catalytic domain suggested that the HD does not serve to block substrate binding, as it does for PARP1. For PARP1, the catalytic domain in the absence of DNA damage is essentially inactive, and deleting the HD (CATΔHD) leads to robust catalytic activity (18). We confirmed this observation using an assay that monitors the automodification activity that most PARP enzymes exhibit (Figure 2A, B). In contrast to PARP1, the PARP4 catalytic domain was clearly active, consistent with the structural observation of an open and accessible active site. Deleting the HD of PARP4 led to an increase in catalytic activity relative to the complete catalytic domain, suggesting that the HD does have the capacity to regulate catalytic output, although not acting as a complete block to substrate access. This activity assay was performed using 5 mM NAD^+^, a relatively high concentration, but under these elevated conditions PARP1 CAT appears essentially inactive and resistant to the utilization of NAD^+^. To test whether the observed PARP4 CAT activity was due largely to the elevated levels of NAD^+^, we repeated the experiment using 100 μM NAD^+^, a concentration that is in agreement with the reported PARP4 *K*_M_ for NAD^+^ (33). Consistent with the observation made using 5 mM NAD^+^, experiments performed using 100 μM NAD^+^ clearly indicated PARP4 CAT domain activity (Figure S2A,B), consistent with an open HD conformation.

To further assess substrate access to the catalytic domain, we used differential scanning fluorimetry to analyze PARP1 and PARP4 catalytic domain interaction with benzamide adenine dinucleotide (BAD), a non-hydrolyzable NAD^+^ analog. An increase in the relative thermal stability of the proteins in the presence of BAD was interpreted as evidence of stabilizing interactions due to ligand binding. As previously reported, the catalytic domain of PARP1 does not appear to interact with BAD, but deletion of the HD removes the substrate block and allows binding to occur (Figure 2C) (21). In contrast, the PARP4 catalytic domain showed evidence of interaction with BAD, and PARP4 with the HD deleted also showed evidence of an interaction. It is notable that the magnitude of the change in relative thermal stability is not necessarily indicative of the strength of the interaction, in particular for proteins with different baseline stabilities. For both PARP1 and PARP4, deletion of the HD lowered the relative stability of the proteins, and the large changes in relative thermal stability upon interaction with BAD for the HD deletions could reflect that these relatively unstable proteins benefit more from the stabilizing forces of the ligand. Thus, we simply used any significant change in relative thermal stability as evidence of an interaction, without quantitatively analyzing the magnitude of the change.

The biochemical analysis of PARP4 catalytic domain activity and substrate binding are thus consistent with the structural observation of an open active site. We conclude that the PARP4 HD does not serve as a strict auto-inhibitory domain, in contrast to its role in PARP1, PARP2 and PARP3. However, the HD of PARP4 is located proximal to the active site and in a position that could influence substrate access and thereby serve an autoregulatory role.

**Figure 3.**
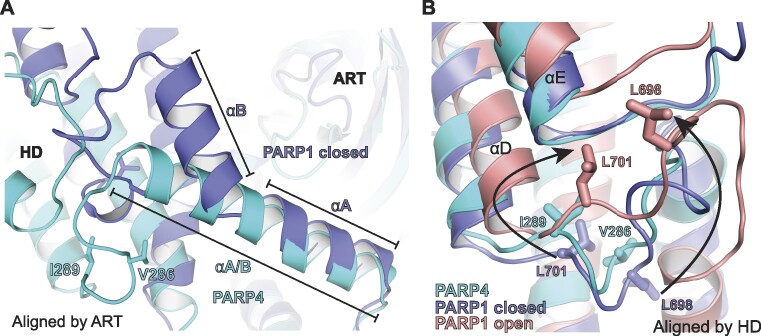
Structural comparison of PARP1 and PARP4 HD conformations. (**A**) Comparison of PARP1 CAT in the closed conformation (drawn in dark blue; PDB: 7AAA) (22) to the PARP4 CAT (drawn in cyan; PDB: 8SX2). The structures were aligned by the ART, with a rmsd of 0.93 Å over 821 atoms. There is an angle between helix αA and helix αB of PARP1, whereas in PARP4 the equivalent region forms a single, continuous and straight helix labeled αA/B. This divergence in structure underlies the differences in the positioning of the HD relative to the ART in PARP1 and PARP4. (**B**) Comparison of PARP1 CAT in the closed conformation (drawn in dark blue; PDB: 7AAA), PARP1 CAT in the open conformation (drawn in pink; PDB: 7S6M) (34), and PARP4 CAT (drawn in cyan, PDB: 8SX2). The structures were aligned by the HD, with a rmsd of 2.2 Å over 486 atoms for PARP1 CAT closed and PARP4 CAT. PARP1 residues L698 and L701 (the ‘leucine switch’) transition from the interior of the HD in the closed conformation to the exterior of the HD fold in the open conformation (highlighted by arrows). The equivalent residues of PARP4, V286 and I289, are positioned in the interior of the HD fold, despite the catalytic domain adopting the open conformation.

The open and accessible conformation of the PARP4 catalytic domain was surprising given that structures of the PARP1 catalytic domain consistently adopt the closed, substrate-blocking conformation. PARP1 requires interaction with DNA damage and major HD structural changes to achieve the open, uninhibited state (18,34). A notable difference between the PARP1 and PARP4 structures is that two helices αA and αB of PARP1 are merged into one continuous helix in PARP4 (Figure 3A; referred to as αA/B in PARP4). Helix αB is one of the most dynamic parts of the PARP1 HD during allosteric activation by DNA strand breaks, where it is repositioned as part of a major conformational transition to open the PARP1 active site (18,34). The presence of the continuous helix αA/B in PARP4 is likely to hold the HD at a specific distance from the ART, essentially maintaining the catalytic region in an open state. The major structural transition in the PARP1 HD also involves the re-positioning of ‘leucine switch’ residues (L698 and L701) from the HD interior toward an interface formed with the regulatory WGR domain (18,34) (Figure 3B). The analogous residues in PARP4 (V286 and I289) remain in the interior of the HD fold, suggesting that PARP4 is held in an open and active conformation without requiring the major unfolding event observed in PARP1.

The PARP4 structures also highlight an N-terminal helix (residues 242 to 255) that exists outside the core fold of both the HD and the ART (Figure S3A). In the collection of PARP4 structures determined in this study, the N-terminal helix adopts several conformations relative to the rest of the HD/ART folds, suggesting that it is flexibly attached to the catalytic domain (Figure S3A). Deletion of this helix did not have an impact on the automodification activity of the catalytic domain (Figure S3B and C), but interestingly, we were not able to crystallize this truncated version of the catalytic domain, suggesting that it contributes in some way to protein stability or dynamics, or perhaps simply aids in forming contacts that allow crystallization.

### Crystals structures of the PARP4 ART domain

**Figure 4.**
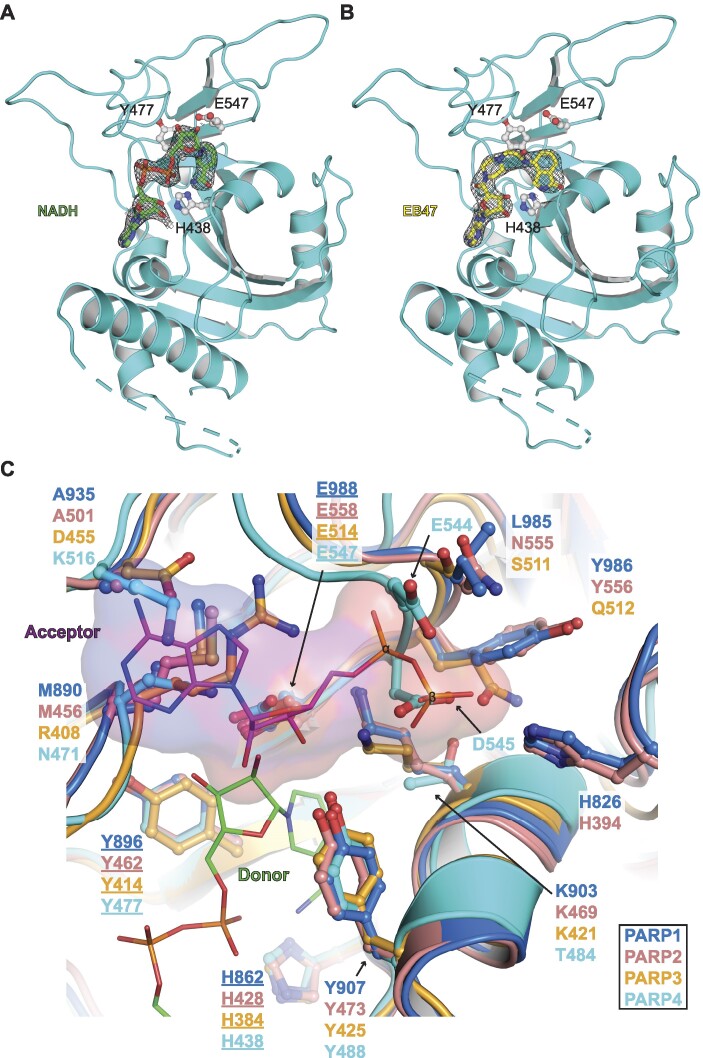
Structural basis for PARP4 MARylation activity. (**A**) Crystal structure of the PARP4 ART domain (drawn in cyan) bound to NADH (drawn as sticks with green carbons) at 2.55 Å resolution (PDB: 8SWY; from this work). Residues of the catalytic triad are drawn as sticks and labeled (H438, Y477, E547). The weighted 2*F*_O_– *F*_C_ electron density map contoured at 1σ is overlayed in the region surrounding the ligand. (**B**) Crystal structure of PARP4 ART domain (drawn in cyan) bound to EB47 (drawn as sticks with yellow carbons) at 3.0 Å resolution (PDB: 8SWZ; from this work). Residues of the catalytic triad are shown as sticks and labeled (H438, Y477, E547). The weighted 2*F*_O_– *F*_C_ electron density map contoured at 1.5σ is overlayed in the region surrounding the ligand. (**C**) Alignment of the ART domain of chicken PARP1 (PDB: 1A26) (35), human PARP2 (PDB: 4PJV) (49), human PARP3 (PDB: 3FHB) (50) and human PARP4 (PDB: 8SWY; from this work). The color code for the structures is shown in the box at the bottom right. The chicken PARP1 catalytic domain was crystallized in complex with carba-NAD^+^, but only the ADP portion of the molecule was visible and modeled (ADP is drawn as sticks with purple carbons and a semi-transparent surface). The ADP binding site defines the ‘acceptor’ site (labeled ‘Acceptor’ in the panel), where the ends of poly(ADP-ribose) chains can dock to be extended by an NAD^+^ molecule bound in the ‘donor’ site (labeled ‘Donor’ in the panel). In this image, the ‘donor’ site is occupied by an NADH molecule (drawn as sticks with green carbons) from the PARP4 ART/NADH structure (panel A). The side chains of residues surrounding the ‘acceptor’ site are labeled and described in the main text. The labels for the HYE catalytic triad residues are underlined; these residues are well-aligned for all of the structures.

We also determined structures of the PARP4 ART domain alone (CATΔHD), which provided higher-resolution insights into the active site configuration that gives rise to PARP4 mono(ADP-ribosylation) activity. The HD was replaced with an 8-residue linker (GSGSGSGG) connecting L282 to S365. CATΔHD crystallized in complex with EB47, and in complex with NADH, the non-hydrolysable reduced form of NAD^+^ (Figure 4A, B). The CATΔHD/NADH crystals diffracted to 2.55 Å and belong to space group C222_1_ with three PARP4 molecules in the asymmetric unit (Table 1). The structure was determined by molecular replacement using a truncated version of the PARP4 CAT/EB47 structure with the ligand removed. Two PARP4 molecules had NADH in their active sites, whereas the active site of the third molecule was empty due to a nearby crystal contact. The structure was refined to a crystallographic *R*/*R*_FREE_ of 0.211/0.254. The CATΔHD/EB47 crystals diffracted to 3.0 Å and belonged to space group *C*222_1_ with three PARP4 molecules in the asymmetric unit (Table 1). Again, only two PARP4 molecules contained EB47 in the active site, due to a crystal contact in the active site of the third PARP4 molecule. Structure refinement yielded a crystallographic *R*/*R*_FREE_ of 0.203/0.248.

### Structural parameters restricting PARP4 to MARylation activity

The structure of the PARP4 ART domain bound to NADH was compared to the closely related ART domains of PARP1, PARP2 and PARP3 in order to investigate the structural basis for the MARylation activity of PARP4 (Figure 4C). Each of these ART domains bear the H-Y-E motif, yet PARP1 and PARP2 exhibit PARylation activity whereas PARP3 and PARP4 are considered MARylation enzymes (12). The relative positions of the H-Y-E residues are very similar between these PARPs, indicating that alteration to H-Y-E positions, in particular the catalytic glutamate, do not underlie PAR versus MAR activity. We turned our attention to the ‘acceptor’ site first identified in PARP1. A chicken PARP1 catalytic domain structure in complex with an NAD^+^ analog provided insight into how ADP-ribose modifications might be extended to form PAR (35). In this structure, the ADP portion of the NAD^+^ analog formed an interaction surface adjacent to the main active site where NAD^+^ is hydrolyzed. This so-called acceptor site is proposed to represent a binding site for an existing ADP-ribose modification to be extended using NAD^+^ from the ‘donor’ site (i.e. the main NAD^+^ binding site conserved in all PARPs) (Figure 4C). Indeed, mutations to key residues in this binding site have an influence on PAR production (35). We inspected the acceptor site of PARP4 relative to PARP1/2/3 to gauge whether alterations to the acceptor site might underlie the MARylation activity of PARP4. As detailed below, our analysis indicates that multiple alterations to the PARP4 acceptor site have rendered it non-functional.

In the acceptor site, main chain atoms from residues L985 and Y986 in PARP1 interact with the α-phosphate of ADP, and the equivalent residues E544 and D545 of PARP4 are positioned such that they would clash with both the α- and β-phosphates. Indeed, this region of the PARP4 ART domain deviates substantially from PARP1/2/3. In addition to the overall position of this region of the structure, the negative charge of the D545 side chain should also interfere with the binding of phosphates in the acceptor site molecule. Moreover, residue K903 in PARP1 interacts with the α-phosphate, and this interaction is conserved in PARP2 and PARP3 (K469 and K421, respectively). In contrast, PARP4 bears T484 at the equivalent position. Notably, mutation of PARP1 residue K903 and PARP2 residue K469 to alanine converts their catalytic output from PARylation to MARylation (36). It is also noteworthy that the PARP4-specific inhibitor AEP07 was developed to target this difference in PARP4 that distinguishes it from the other H-Y-E PARPs (37).

Another acceptor site interaction is mediated by residue H826 of PARP1 (H394 in PARP2). H826 engages the β-phosphate of ADP in the acceptor site, and the corresponding loop in PARP4 is shorter and essentially lacks an equivalent residue at this position. PARP3 also has a shortened loop and lacks a residue equivalent to H826 in PARP1. Mutagenic analysis of residue H826 in PARP1 and residue H394 in PARP2 support their role in mediating PARylation activity (38,39), thus the lack of this key residue in PARP4 is likely a driving factor in the lack of PARylation activity.

There are also changes in the interactions surrounding the adenine base of ADP in the acceptor site. The adenine base of ADP rests against M890 in PARP1 (M456 in PARP2), whereas the equivalent position in PARP4 is N471 and in PARP3 is R408. The polar residues N471 and R408 are unlikely to serve the same specific role as the methionines in PARP1/2, although there are potential conformations of these residues that could potentially support interaction with the adenine base. Furthermore, the charged residue K516 of PARP4 (D455 in PARP3) extends into this area of the acceptor site, whereas PARP1 and PARP2 have an alanine at this position. Together, these alterations are expected to prevent the interaction with the adenine base in the acceptor site. An alignment of all of the ART structures determined in this study did not provide any evidence of flexibility in the acceptor site region of PARP4 (Figure S4A,B). This analysis suggests that the positioning of residues surrounding the acceptor site region are largely static.

Collectively, these alterations in PARP4 should strongly disfavor the binding of the phosphates in the acceptor site, and also the adenine base to a certain extent. We thus conclude that PARP4 does not maintain a functional acceptor site as defined by the chicken PARP1 structure. The disruptive alterations that we observe in our crystal structures are consistent with the reported MARylation activity of PARP4.

### BRCT and WGR domains downregulate PARP4 catalytic domain activity

**Figure 5.**
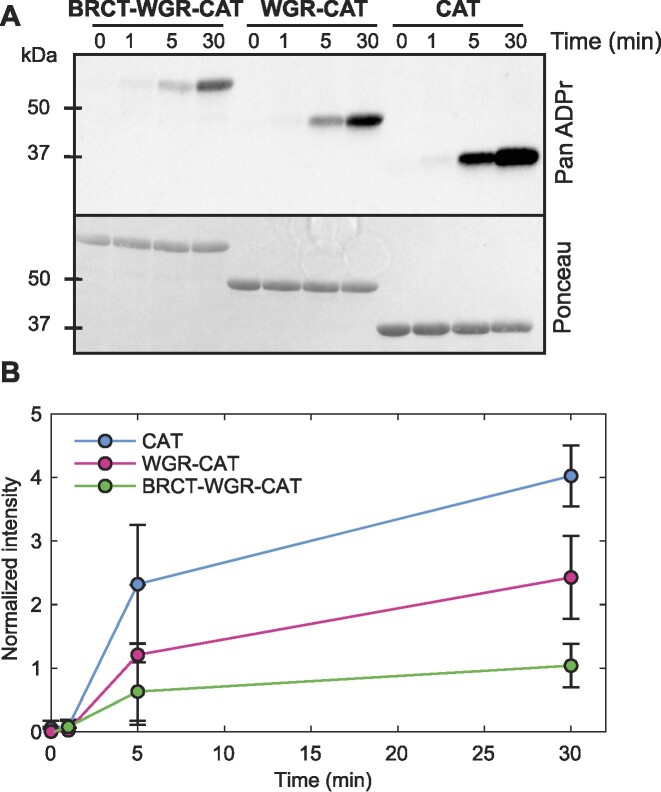
The BRCT and WGR domains downregulate PARP4 MARylation activity. (**A**) Western blot catalytic activity assay monitoring PARP4 automodification. The BRCT–WGR–CAT, WGR–CAT and CAT fragments (2 μM) were individually incubated in the presence of 5 mM NAD^+^ for the indicated amount of time. Protein modification was detected with a pan-ADP-ribose binding reagent, and Ponceau staining indicated the amount of protein included in each sample. This is a representative experiment that has been repeated three times. (**B**) Quantification of the three repeats of the experiment shown in panel A. The PanADPr intensity was normalized to the Ponceau intensity measurements. The averages and standard deviations are plotted.

Given the common BRCT–WGR–CAT domain organization in PARP1 and PARP4, we extended our study beyond the catalytic region to analyze the catalytic output and nucleic acid binding activity of the BRCT–WGR–CAT region of PARP4. In terms of catalytic output, the BRCT–WGR–CAT of PARP4 reproducibly exhibited lower activity relative to the catalytic domain alone, with a ∼3.7-fold reduction in automodified band intensity at the 5-minute timepoint (Figure 5A, B). We also created the WGR–CAT construct, in order to test whether the BRCT domain alone was responsible for the decrease in activity relative to CAT. The WGR–CAT construct exhibited a level of activity higher than the BRCT–WGR–CAT construct (∼1.9-fold higher). However, the activity of the WGR–CAT fragment was still lower than the CAT domain alone (∼1.9-fold lower). Similar to the previous activity assay presented in this study, we performed these activity assays at two different NAD^+^ concentrations (5 mM in Figure 5A, B; 100 μM in Figure S2C, D). The same trend was observed in both conditions, although the signal was overall weaker when using the lower NAD^+^ concentration. The analysis of PARP4 N-terminal fragments thus suggested that the BRCT and WGR domains collectively lower PARP4 activity. We can anticipate that the BRCT and WGR domains form contacts with the CAT to restrict catalytic output, through a currently unknown mechanism, but potentially through contacts with the HD as seen in PARP1. Indeed, the AlphaFold analysis of human PARP4 indicates potential contacts between the WGR and CAT regions (Figure S5A,B).

### PARP4 BRCT–WGR–CAT binds to vtRNA

PARP1 is well known for its capacity to bind DNA breaks, and the WGR domain is an integral component of the interaction with DNA breaks. PARP1 also engages undamaged DNA, and the BRCT domain has recently been identified as a region that engages continuous DNA duplex. Moreover, PARP1 is also expected to be regulated through interaction with RNA (40–43), although the molecular details of the interactions are not yet understood. Given that the BRCT and WGR domains of PARP1 are both involved in nucleic acid interactions, we expected that these domains in PARP4 might play similar roles. The localization of PARP4 and vtRNA within vault particles also compelled us to ask whether these two components interact.

**Figure 6.**
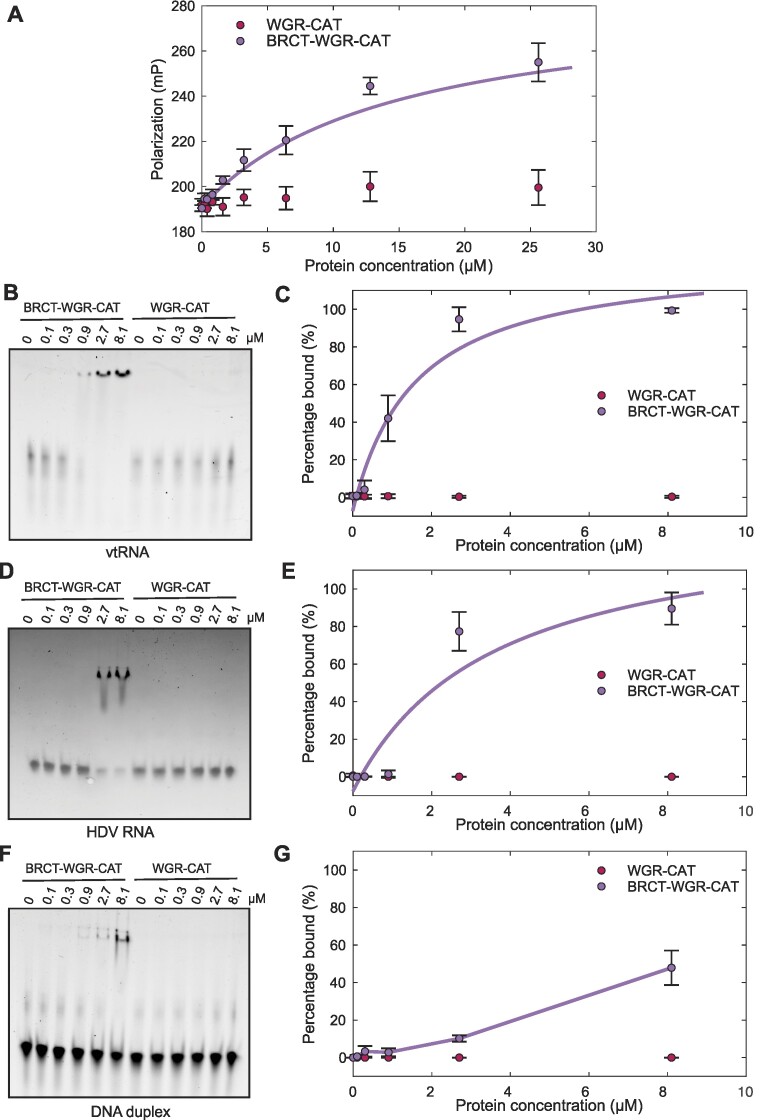
The BRCT domain of PARP4 binds selectively to vtRNA. (**A**) FP binding assay using 30 nM vtRNA bearing a fluorescent label. The BRCT–WGR–CAT and WGR–CAT fragments were incubated with vtRNA at the designated concentrations. The data points and error bars represent the average and standard deviation of three independent experiments. A 1:1 binding model was fit to the BRCT–WGR–CAT binding curve, yielding an apparent *K*_D_ of 15 ± 9 μM. WGR–CAT did not show evidence of binding at these concentrations. (**B**) EMSA using 100 nM vtRNA bearing a fluorescent label. BRCT–WGR–CAT and WGR–CAT were incubated with vtRNA at the indicated concentrations. This is a representative experiment that has been repeated three times. (**C**) Quantification of the three repeats of the EMSA shown in panel B. The data points and error bars represent the average and standard deviation of three independent experiments. A 1:1 binding model was fit to the BRCT–WGR–CAT binding curve, yielding an apparent *K*_D_ of 1.5 ± 0.4 μM. WGR–CAT did not show evidence of binding at these concentrations. (**D**) EMSA using 100 nM HDV. SYBR Gold staining was used to visualize the RNA. BRCT–WGR–CAT and WGR–CAT were incubated with the RNA at the indicated concentrations. This is a representative experiment that has been repeated three times. (**E**) Quantification of the three repeats of the EMSA shown in panel D. The data points and error bars represent the average and standard deviation of three independent experiments. A 1:1 binding model was fit to the BRCT–WGR–CAT binding curve, yielding an apparent *K*_D_ of 3.4 ± 0.3 μM. WGR–CAT did not show evidence of binding at these concentrations. (**F**) EMSA using 100 nM 47-basepair DNA duplex. SYBR Gold staining was used to visualize the DNA. BRCT–WGR–CAT and WGR–CAT were incubated with the DNA at the indicated concentrations. (**G**) Quantification of the three repeats of the EMSA shown in panel F. The data points and error bars represent the average and standard deviation of three independent experiments. Less than 50% of the DNA was shifted at the highest BRCT–WGR–CAT concentration; therefore, a binding model was not fit to the data and the data points were simply connected to illustrate the trend. WGR–CAT did not show evidence of binding at these concentrations.

As a first analysis of the potential nucleic acid binding properties of BRCT–WGR–CAT of PARP4, we performed fluorescence polarization (FP) and electrophoretic mobility shift assay (EMSA) analysis using a chemically synthesized and fluorescently labeled vtRNA. Using FP, the BRCT–WGR–CAT exhibited reproducible interaction with vtRNA, with an apparent equilibrium binding constant (*K*_D_) of 15 ± 9 μM (Figure 6A). Interestingly, WGR–CAT did not exhibit interaction with vtRNA (Figure 6A), suggesting that the BRCT domain is the major contributor to the observed interaction. Using EMSA, the BRCT–WGR–CAT shifted the migration of vtRNA into a distinct band (Figure 6B), with an apparent *K*_D_ of 1.5 ± 0.4 μM (Figure 6C). We observed that the WGR–CAT fragment was not able to shift the migration of vtRNA over the same concentration range, again suggesting that the BRCT domain is largely responsible for the observed interaction. We anticipate that the difference in affinity observed by FP versus EMSA can at least partly be explained by the well-known ‘caging’ effect of EMSA, where limited mobility in the gel matrix increases the local concentration of the protein. We also tested BRCT–WGR–CAT and WGR–CAT interaction with Hepatitis delta virus (HDV) RNA, which is unrelated to vtRNA but has a similar number of ribonucleotides and a comparable amount of duplex regions in its structure based on Mfold analysis (44) (Figure S6). BRCT–WGR–CAT showed evidence of interaction with HDV RNA in an EMSA analysis (Figure 6D, E). Quantification of the interaction yielded an apparent *K*_D_ of 3.4 ± 0.3 μM, and thus less robust than the interaction observed with vtRNA (*K*_D_ of 1.5 ± 0.4 μM). The WGR–CAT fragment did not bind to HDV RNA, consistent with the observation that the BRCT domain bears the main nucleic acid binding activity.

To assess the specificity of the interaction for RNA versus DNA, we used EMSA to measure interaction with a 47-base pair duplex DNA, which contains a similar number of nucleotides as vtRNA. BRCT–WGR–CAT exhibited a weak interaction with the DNA that was most apparent at the highest concentration of 8.1 μM, at which point roughly 40% of the DNA was bound (Figure 6F, G). For comparison, BRCT–WGR–CAT achieved a similar level of vtRNA binding at 0.9 μM (Figure 6B, C). WGR–CAT did not interact with the DNA (Figure 6F, G). Using EMSA, a 47-nucleotide single-stranded DNA interacted very weakly with BRCT–WGR–CAT at the highest concentration tested (Figure S7A, B). The overall binding assessment allows us to conclude that the BRCT–WGR–CAT fragment has micromolar-level affinity for vtRNA, and that the BRCT domain is primarily responsible for the interaction. There is a measurable level of specificity toward vtRNA over the unrelated HDV RNA. The similar amount of duplex in these two RNA structures could indicate that the BRCT is engaging duplex regions of RNA, but further structural work is needed to understand the BRCT interaction with RNA. BRCT–WGR–CAT exhibited a notable specificity toward RNA versus DNA.

### vtRNA does not modulate the catalytic activity of BRCT–WGR–CAT

**Figure 7.**
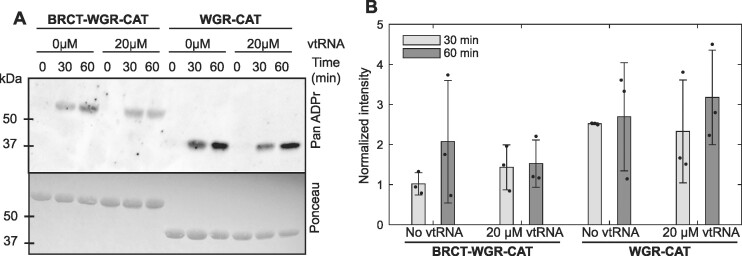
vtRNA does not modulate PARP4 MARylation activity. (**A**) Western blot catalytic activity assay monitoring PARP4 automodification. PARP4 BRCT–WGR–CAT and WGR–CAT (5 μM) were individually incubated in the absence/presence of 20 μM vtRNA and 100 μM NAD^+^ for the indicated time points. Protein modification was detected with a pan-ADP-ribose binding reagent, and Ponceau staining indicated the amount of protein included in each sample. This is a representative experiment that has been repeated three times. (**B**) Quantification of the three repeats of the activity assay shown in panel A. The PanADPr intensity was normalized to the Ponceau intensity measurements. The individual measurements from repeat experiments are plotted. The bars represent the averages and error bars represent standard deviations.

Interaction with DNA strand breaks potently increases PARP1 ADP-ribosylation activity, thus we were interested to investigate whether PARP4 was regulated by the vtRNA interaction that we established. Under conditions where we clearly observed BRCT–WGR–CAT interaction with vtRNA, we did not observe any notable changes in the catalytic output of BRCT–WGR–CAT in the absence versus presence of vtRNA (Figure 7A). Even potential subtle differences in activation levels were deemed unlikely to be mediated by vtRNA interaction, as deletion of the nucleic acid binding BRCT domain (i.e. using WGR–CAT) gave essentially the same results (Figure 7A, B). This experiment again showed the difference in catalytic activity between BRCT–WGR–CAT and WGR–CAT, as also observed in Figure 5A. The activity assay results suggest that the interaction with nucleic acid is not a potent regulator of PARP4, in stark contrast to the acute activation of PARP1 in response to DNA strand break interaction. It is conceivable that other components of PARP4, or the vault particle, are required to enact nucleic acid-dependent regulation, but it is also possible that PARP4 interaction with vtRNA mediates another function.

### Comparison of the BRCT and WGR domains of PARP1 and PARP4

**Figure 8.**
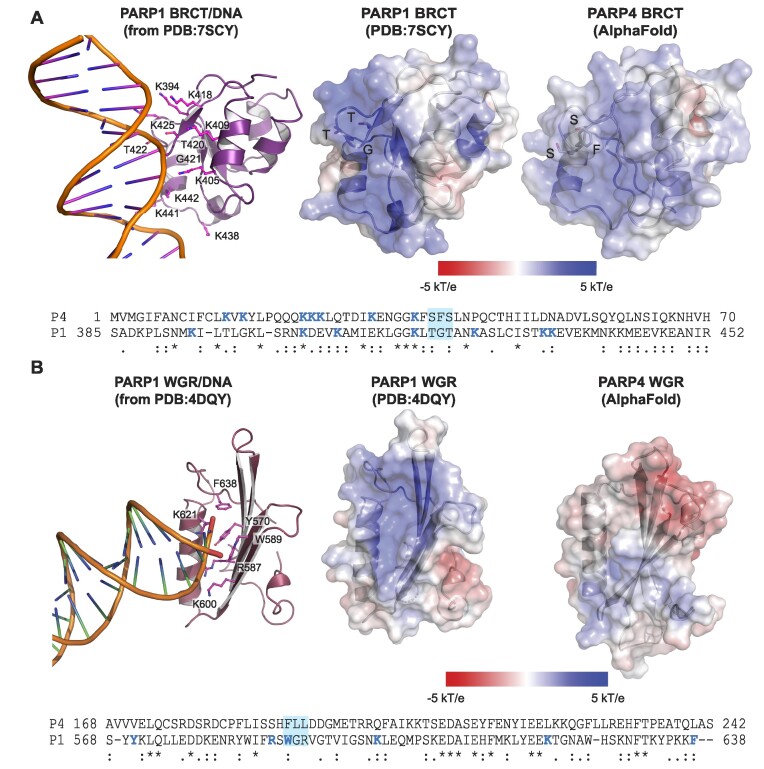
Comparison of the BRCT and WGR domains of PARP1 and PARP4. (**A**) Comparison of the PARP1 and PARP4 BRCT domains. The PARP1 BRCT domain bound to nucleosomal duplex DNA is shown on the left (PDB: 7SCY) (23). The electrostatic surface potential of the PARP1 BRCT from structure 7SCY is shown in the middle in the same orientation. The electrostatic surface potential of the AlphaFold model of PARP4 BRCT domain is shown on the right in the same relative orientation. The PARP1 T-G-T motif and the PARP4 S-F-S motif are shown as sticks and are labeled in the structure models. The coloring scale is displayed, with blue representing electropositive and red representing electronegative. The sequences of the PARP1 and PARP4 BRCT domains are aligned at the bottom of the panel (an asterisk represents sequence identity, double dots represent strong conservation, and single dot represents weak conservation). The lysine residues that contribute to the PARP1 BRCT DNA binding interface (leftmost image) are colored blue in PARP1 (P1). PARP4 (P4) lysine residues in the vicinity of this same surface are also colored blue. The DNA binding T-G-T motif of PARP1 is highlighted with a blue box and corresponds to S-F-S in PARP4. (**B**) Comparison of the PARP1 and PARP4 WGR domains. The PARP1 WGR domain bound to a DNA duplex end is shown on the left (PDB: 4DQY) (20). The electrostatic surface potential of the PARP1 WGR from structure 4DQY is shown in the middle in the same orientation. The electrostatic surface potential of the AlphaFold model of PARP4 WGR is shown on the right in the same relative orientation. The coloring scale is displayed, with blue representing electropositive and red representing electronegative. The sequences of the PARP1 and PARP4 WGR domains are aligned at the bottom of the panel (an asterisk represents sequence identity, double dots represent strong conservation, and single dot represents weak conservation). The residues involved in PARP1 WGR binding to DNA (leftmost panel) are colored blue in the sequence alignment. The characteristic W-G-R residues of the PARP1 WGR domain are highlighted in the blue box, highlighting that these residues are not conserved in PARP4.

To better understand the differences between PARP1 and PARP4 in the BRCT and WGR domains, we compared experimental PARP1 structures for these domains (20,23) to the predicted AlphaFold structures for the PARP4 BRCT and WGR domains (45,46) (Figure 8A, B). The AlphaFold structures are primarily confident to highly confident in the folded regions of BRCT and WGR, and were thus deemed reasonable models for overall structural comparisons (Figure S5). The BRCT and WGR domains of PARP1 have been captured in complex with nucleic acid, thus the analysis allowed us to compare the structures in terms of the regions that engage nucleic acid. As expected, the electrostatic surface potential of the PARP1 BRCT domain exhibits a concentrated, electropositive surface in the region that engages nucleic acid (Figure 8A). The predicted AlphaFold structure for the PARP4 BRCT domain exhibited an electropositive surface in the same region, suggesting that the PARP1 and PARP4 BRCT domains use similar surfaces to engage nucleic acid. However, there are notable sequence differences between PARP1 and PARP4 (Figure 8A). As a notable example, the Thr-Gly-Thr motif highlighted in the structure of PARP1 BRCT engaging nucleosomal duplex DNA (23) corresponds to a Ser-Phe-Ser motif in the BRCT domain of PARP4. Moreover, only a few of the PARP1 BRCT lysine residues confirmed to participate in DNA binding (23) are conserved in PARP4 (Figure 8A). We anticipate that these differences underlie the capacity of the PARP4 BRCT domain to engage RNA structure.

The PARP1 WGR domain exhibited an electropositive surface in the region that interacts with nucleic acid (Figure 8B), as expected. In contrast, the WGR domain of PARP4 did not exhibit a continuous electropositive surface over the same region. In fact, roughly half of the nucleic acid binding surface observed in PARP1 was electronegative in PARP4 (Figure 8B). Consistently, the main nucleic-acid binding residues of PARP1 are not conserved in PARP4 (Figure 8B). Interestingly, not even the namesake Trp-Gly-Arg residues are conserved in PARP4, which instead has Phe-Leu-Leu in place of these residues. This analysis suggests that the WGR surface of PARP4 is not likely to engage nucleic acid in the same manner as PARP1, and perhaps that the WGR of PARP4 does not actually bind nucleic acid. The structural analysis was thus consistent with the vtRNA binding experiments (Figure 6A–C) that indicated that the BRCT domain is the major contributor to nucleic acid interaction.

## Discussion

PARP4 is a distinct member of the PARP family of ADP-ribosyltransferases with a special association with vault particles and seemingly a connection to DNA damage-dependent PARPs in terms of domain composition. This study focused on the PARP1-homology region of PARP4 to address unanswered questions on biochemical and structural aspects of PARP4. Analysis of the PARP4 catalytic region uncovered the active site arrangement that restricts PARP4 to MARylation or mono(ADP-ribosylation) activity, with several adjustments relative to PARP1 and PARP2 that yield an inactive acceptor site (Figure 4C). While it is clear that the PARP4 catalytic region on its own lacks the capacity to produce poly(ADP-ribose), there still exists the possibility that other factors could potentially regulate PARP4 output, akin to HPF1 regulation of PARP1 and PARP2 (38). PARP4 does not have the structural features required for HPF1 interaction.

The PARP4 catalytic domain crystal structure represents an ‘open’ conformation that is capable of binding substrate NAD^+^, based on the complex with the NAD^+^-mimic EB47 and the binding analysis with the non-hydrolyzable NAD^+^ analog BAD. In contrast, crystal structures of the PARP1 catalytic domain are routinely observed in a ‘closed’ conformation, in which the HD adopts a conformation that blocks NAD^+^ binding. The ‘open’ conformation of PARP1 has only been captured in the presence of the DNA-break binding regulatory domains (Zn1, Zn3, WGR), and this conformation requires major remodelling of the HD relative to the ‘closed’ conformation, including a partial unfolding of the HD core fold. Our analysis indicates that PARP4 achieves the ‘open’ conformation while maintaining the core fold of the HD. We expect that the continuous αA/B helix is the key structural element that holds PARP4 in the open conformation. Deletion mutagenesis suggests that the HD is still positioned to influence NAD^+^ access, as the removal of the HD led to an increase in catalytic activity (Figure 2A). Future studies will focus on mechanisms that could potentially modulate HD conformation, as it is still possible that the PARP4 HD will adopt different states.

In addition to the catalytic domain (HD and ART), PARP4 and PARP1 homology includes a BRCT and WGR domain. In PARP1, the WGR domain is essential for PAR catalysis in response to DNA strand breaks, and the BRCT domain engages continuous duplex DNA. The BRCT–WGR–CAT region of PARP4 exhibited an interaction with vtRNA, and the BRCT domain appears to be the primary driver of the interaction. However, the vtRNA interaction did not have an apparent regulatory effect on the catalytic output of PARP4. While it is possible that other domains or proteins factors are required for PARP4 to respond to vtRNA, it is also possible that the vtRNA interaction mediates a different function. For example, interaction with vtRNA could serve to appropriately position PARP4 within vault, after the MVP-ID domain localizes PARP4 to the vault. Although PARP4 exhibited a modest micromolar affinity for vtRNA, the co-localization of these two components within the vault could drive a high local concentration and promote their interaction. BRCT–WGR–CAT and WGR–CAT exhibited lower catalytic output relative to the CAT domain of PARP4. These results strongly suggest that these domains regulate catalysis through some mechanism that is likely to be allosteric and potentially mediated through the HD, perhaps pushing the HD into a more ‘closed’ state. The AlphaFold model of PARP4 indeed predicts WGR interactions with the catalytic domain, and future studies will hopefully capture the details and consequences of the potential interdomain interactions of PARP4.

In conclusion, our study of PARP4 provides new insights into the regulation of PARP family enzymes, and the variation that exists even between closely related members of the family. Our continuing work focuses on further defining the distinct mechanisms of PARP4 regulation.

## Supplementary Material

gkad1064_Supplemental_File

## Data Availability

The atomic coordinates and structure factors have been deposited in the Protein Data Bank (https://www.rcsb.org) and are publicly available as of the date of publication. Accession codes are PDB: 8SWY (PARP4 CATΔHD bound to NADH), PDB: 8SWZ (PARP4 CATΔHD bound to EB47), PDB: 8SX1 (PARP4 catalytic domain), and PDB: 8SX2 (PARP4 catalytic domain bound to EB47). Any additional information required to reanalyze the data reported in this paper is available from the corresponding author upon request.
