# Supplementary material for: Structural and biochemical analysis of the PARP1-homology region of PARP4/vault PARP
Source: Nucleic Acids Res. 2023 Nov 16;51(22):12492–507. doi: 10.1093/nar/gkad1064 (PMC10711553; doi:10.1093/nar/gkad1064)
Supplement: gkad1064_Supplemental_File [file gkad1064_supplemental_file.pdf]

Supplementary Material

Structural and biochemical analysis of the PARP1-homology region of PARP4/vault PARP

Léonie Frigon and John M. Pascal

Content:

Figure S1. Activity assays showing the linear range of the automodification signal.

Figure S2. Activity assays at lower NAD^+ concentration and linearity experiments.

Figure S3. Analysis of the impact of N-terminal helix deletion

Figure S4. Analysis of PARP4 acceptor site flexibility

Figure S5. AlphaFold analysis of PARP4 structure

Figure S6. Secondary structures of vtRNA and HDV RNA

Figure S7. EMSA analysis of PARP4 interaction with DNA

Figure S1.

A

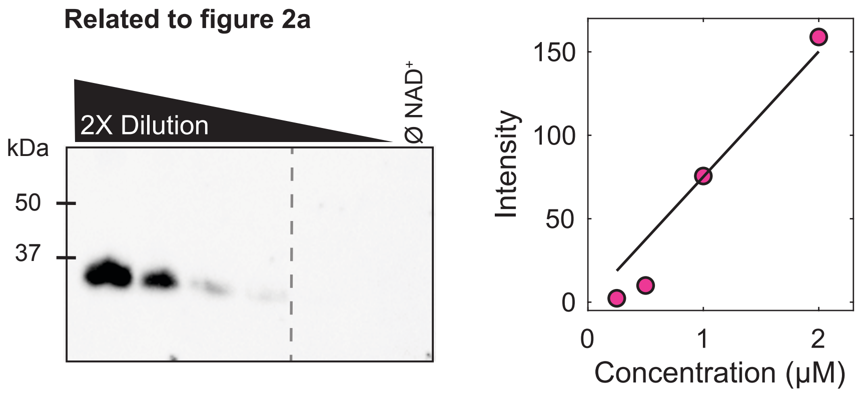

Related to figure 2a
2X Dilution
Ø NAD+
kDa
50
37
Intensity
150
100
50
0
0
1
2
Concentration (µM)

B

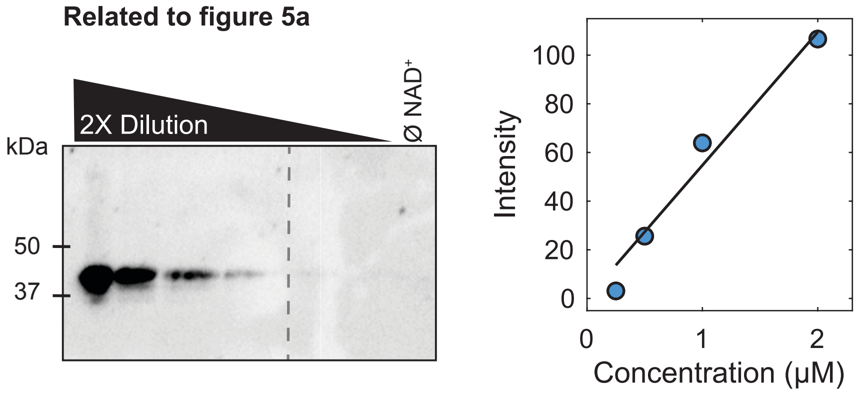

Related to figure 5a
2X Dilution
Ø NAD+
kDa
50
37
Intensity
100
80
60
40
20
0
0
1
2
Concentration (µM)

Figure S1. Evaluation of the linearity of the signal in the Western blot activity assay.

A. Western Blot analysis of PARP4 CATΔHD automodification (related to main Figure 2A). (left image) PARP4 CATΔHD (2 μM) was incubated for 60 minutes with 5 mM NAD^+, the highest signal measured in this analysis. This reaction and a series of 2-fold dilutions of the reaction were then evaluated. Dilutions of the sample on the right side of the dashed line were essentially at background level, which was a no NAD^+ sample (labeled as ∅ NAD^+). (right plot) The measurements on the left side of the dashed line in the image were plotted versus the concentration of PARP4 in the sample (0.25, 0.5, 1, and 2 μM). These dilutions approximated a linear relationship (R-square of 0.93), indicating that the intensity measurements over this 8-fold dilution range can be considered linear. The measurements presented in Figure 2A are within this linear range of detection.

B. Western Blot catalytic activity assay measuring PARP4 CAT automodification (related to Figure 5A). (left image) The CAT fragment (2 μM) was incubated 30 minutes with 5 mM NAD^+, the highest signal measured in the Figure 5A analysis. This reaction and a series of 2-fold dilutions of the reaction were then evaluated. Dilutions of the sample on the right side of the dashed line were essentially at background level, which was a no NAD^+ sample (labeled as ∅ NAD^+). (right plot) The measurements on the left side of the dashed line in the image were plotted versus the concentration of PARP4 in the sample (0.25, 0.5, 1, and 2 μM). These dilutions approximated a linear relationship (R-square of 0.97), indicating that the intensity measurements over this 8-fold dilution range can be considered linear. The measurements presented in Figure 5A are within this linear range of detection.

Figure S2.

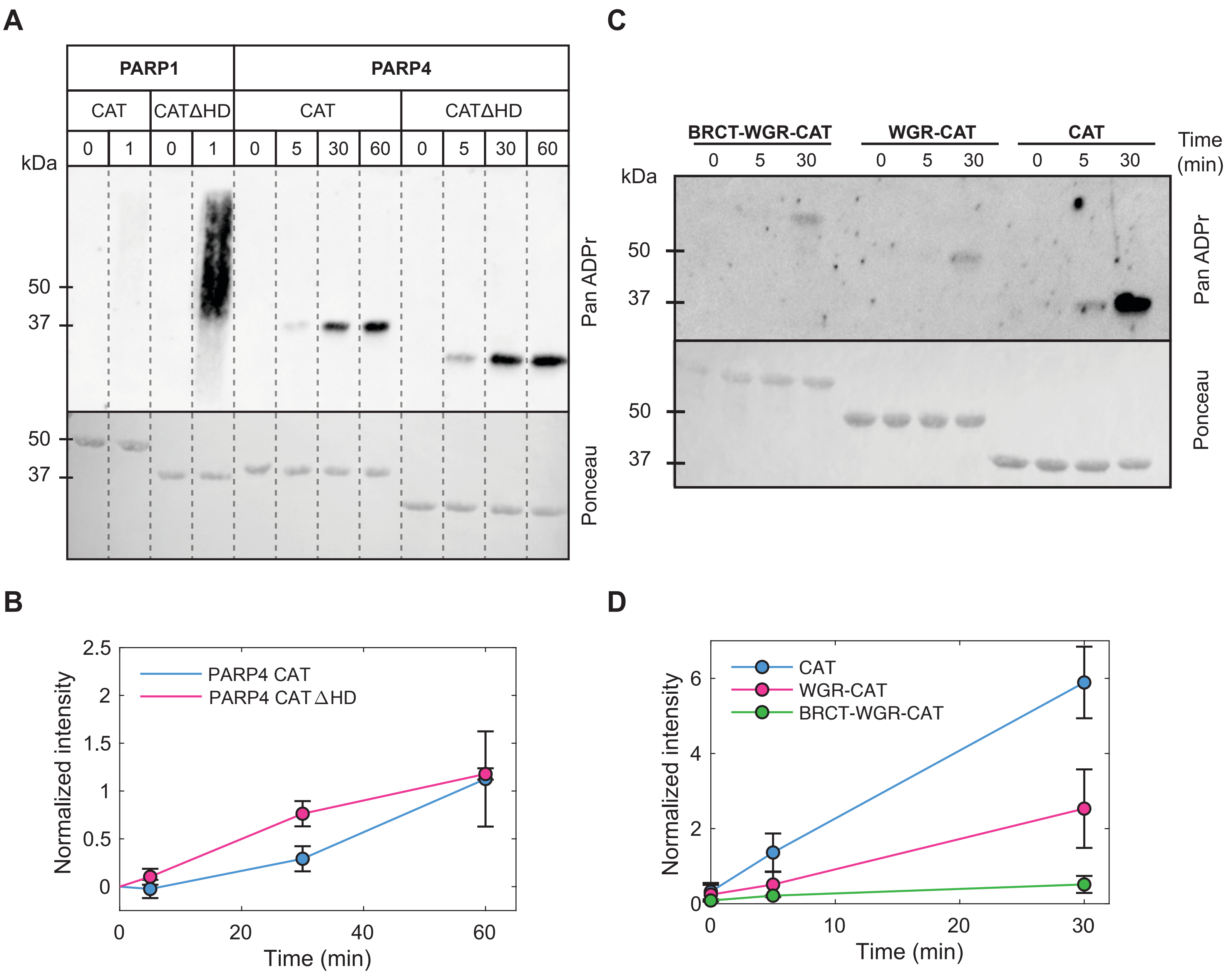
A
PARP1
PARP4
CAT
CATΔHD
CAT
CATΔHD
0 1 0 1 0 5 30 60 0 5 30 60
kDa
50
37
50
37
Pan ADPr
Ponceau
B
Normalized intensity
Time (min)
PARP4 CAT
PARP4 CAT ΔHD
C
BRCT-WGR-CAT
WGR-CAT
CAT
Time (min)
0 5 30 0 5 30 0 5 30
kDa
50
37
50
37
Pan ADPr
Ponceau
D
CAT
WGR-CAT
BRCT-WGR-CAT
Normalized intensity
Time (min)

Figure S2. PARP4 activity assays using 100 μM NAD^+

A. Western Blot catalytic activity assay measuring PARP1/PARP4 automodification. Purified proteins (2 μM) were incubated with 100 μM of NAD^+ for the indicated time points. A pan-ADP-ribose binding reagent was used for detection of modified proteins. Ponceau staining revealed the amount of protein in each reaction. A representative experiment is shown. The experiment was performed three times.

B. Quantification of the three repeats of the experiment shown in panel A. The PanADPr intensity was normalized to the Ponceau intensity measurements. The averages and standard deviations are plotted.

C. Western blot catalytic activity assay monitoring PARP4 automodification. The BRCT-WGR-CAT, WGR-CAT, and CAT fragments (2 μM) were individually incubated in the presence of 100 μM NAD^+ for the indicated amount of time. Protein modification was detected with a pan-ADP-ribose binding reagent, and Ponceau staining indicated the amount of protein included in each sample. This is a representative experiment that has been repeated three times.

D. Quantification of the three repeats of the experiment shown in panel C. The PanADPr intensity was normalized to the Ponceau intensity measurements. The averages and standard deviations are plotted.

Figure S3.

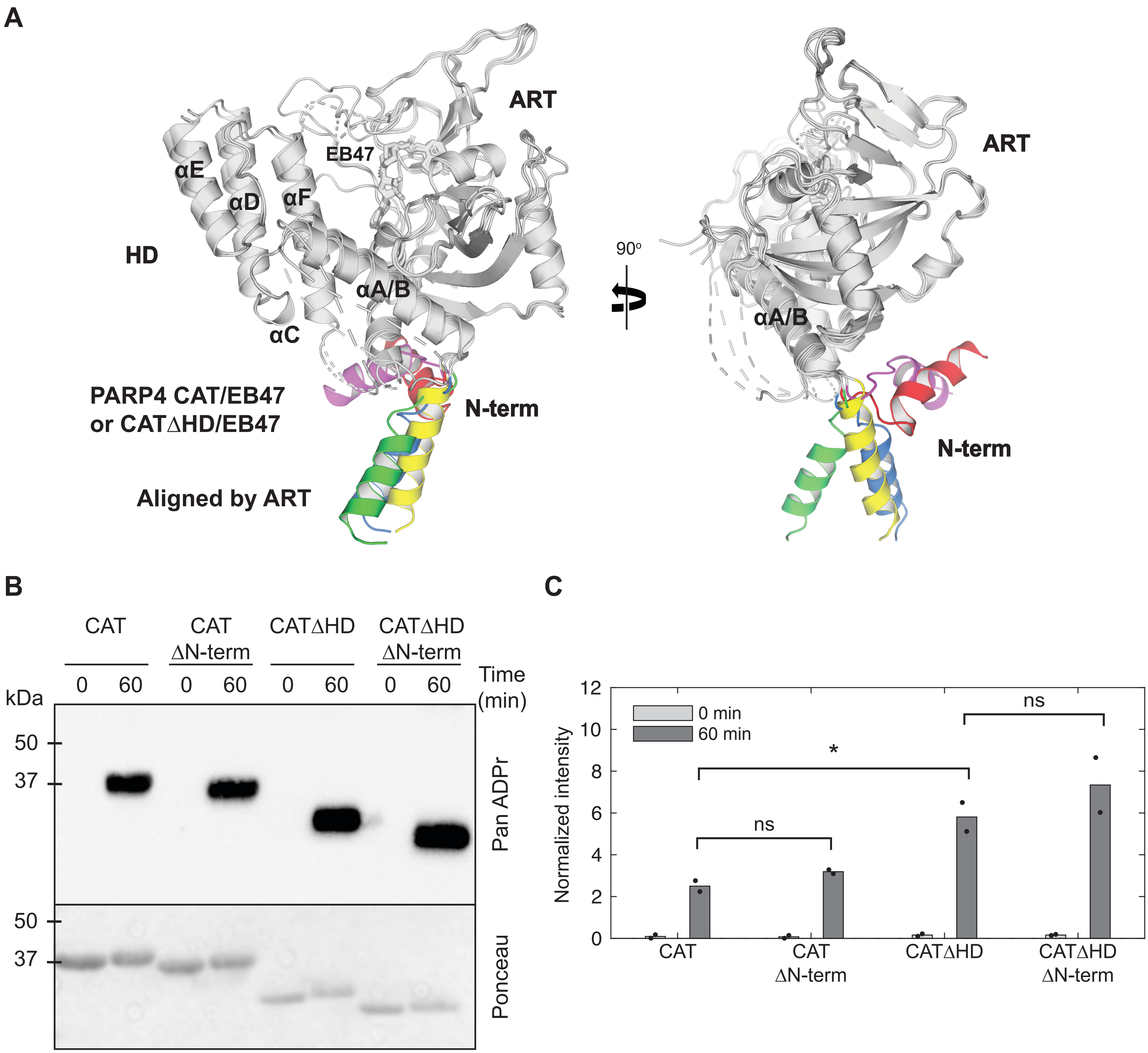
A
ART
EB47
αE
αD
αF
HD
αA/B
αC
PARP4 CAT/EB47
or CATΔHD/EB47
N-term
Aligned by ART
90°
ART
αA/B
N-term
B
CAT
CAT ΔN-term
CATΔHD
CATΔHD ΔN-term
0
60
Time (min)
kDa
50
37
Pan ADPr
Ponceau
C
0 min
60 min
Normalized intensity
12
10
8
6
4
2
0
ns
*
ns
CAT
CAT ΔN-term
CATΔHD
CATΔHD ΔN-term

Figure S3. Analysis of the impact of N-terminal helix deletion

A. Structural alignment of 2 non-crystallographic symmetry copies of the PARP4 CAT in complex with EB47, and 3 non-crystallographic symmetry copies of CATΔHD in complex with EB47. The structures are aligned by their ART fold and show the variable position of the N-terminal helix (colored in purple, red, green, blue, and yellow) relative to the rest of the structure. There are two views of the structural alignment that differ by a 90° rotation.

B. Western Blot catalytic activity assay measuring PARP4 CAT and CATΔHD automodification in presence or absence of the N-terminal helix. Purified PARP4 fragments (2 μM) were incubated in the presence of 5 mM NAD^+ for the indicated time points. A pan-ADP-ribose binding reagent was used for detection of modified proteins. Ponceau staining revealed the amount of protein in each reaction.

C. Quantification of the two repeats of the assay shown in panel B. The PanADPr intensity was normalized to the Ponceau intensity. The individual measurements are plotted. The bar represents the average of the two measurements. A two-sample two-sided t-test was used to compare the indicated samples. *, $p < 0.05$; ns, not significant.

Figure S4.

A

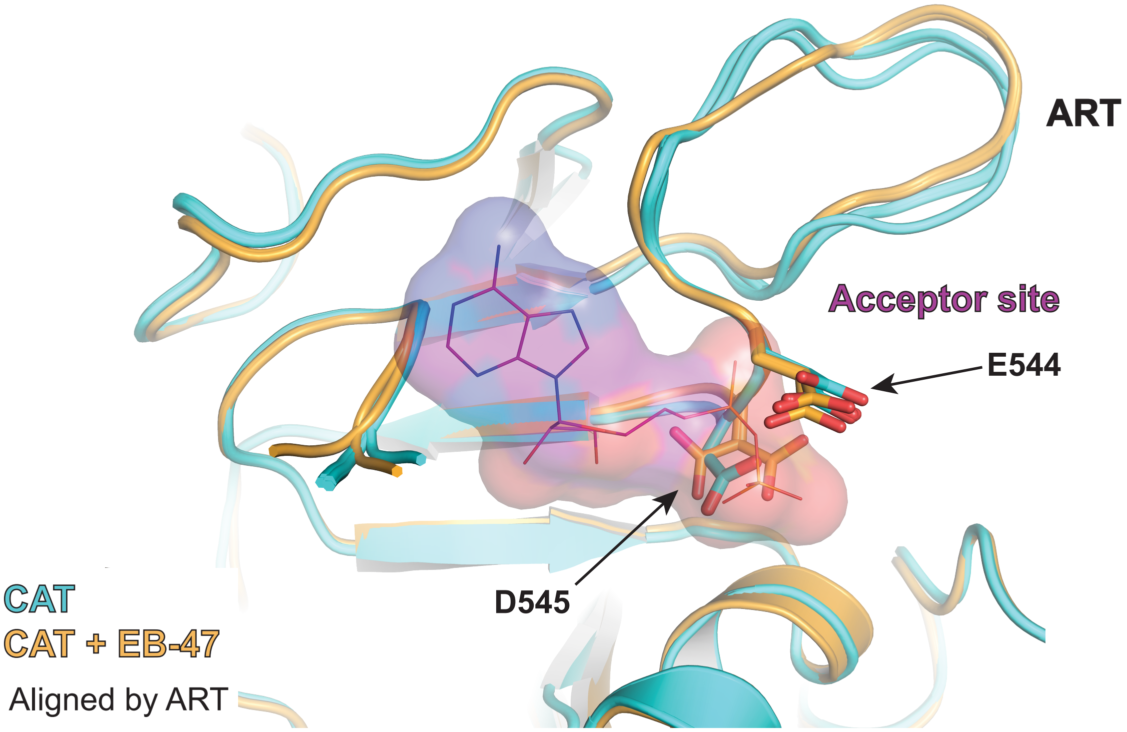
ART
Acceptor site
E544
D545
CAT
CAT + EB-47
Aligned by ART

B

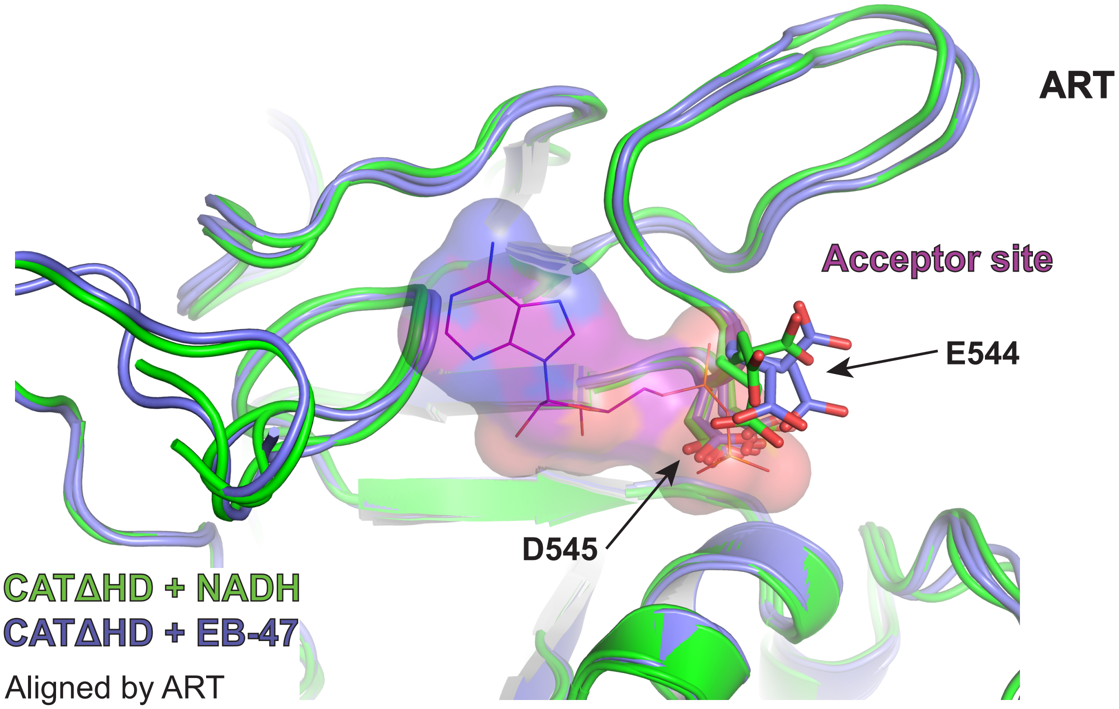
ART
Acceptor site
E544
D545
CATΔHD + NADH
CATΔHD + EB-47
Aligned by ART

Figure S4. Analysis of PARP4 acceptor site flexibility

A. Alignment of the PARP4 CAT domain structures determined in this study (10 copies), with the models colored based on the crystal structure from which they are derived (CAT or CAT/EB47). The Acceptor site is defined by the structure of carba-NAD^+ bound to chicken PARP1 (PDB: 1A26) [35]. The ADP portion of carba-NAD^+ was modeled in 1A26, and these coordinates are shown as sticks and a surface to highlight the acceptor site. The PARP4 structures are consistent in the region of the acceptor site, suggesting that these regions are not flexible. Residues E544 and D545, which are positioned to interfere with the phosphates of ADP, are drawn as sticks. The main chain atoms of these residues are quite consistent between the different structures and always in a position to interfere with ADP binding. There is some mobility of the side chain atoms, but they all still appear to be in a position to cause an electrostatic repulsion with the phosphates.

B. Alignment of the PARP4 CATΔHD domain structures determined in this study (6 copies). The models are colored based on the crystal structure from which they are derived (CATΔHD/NADH and CATΔHD/EB47). The ADP molecule is shown as described in panel A. The structures are consistent in the region of the Acceptor site, similar to the analysis in panel A.

Figure S5.

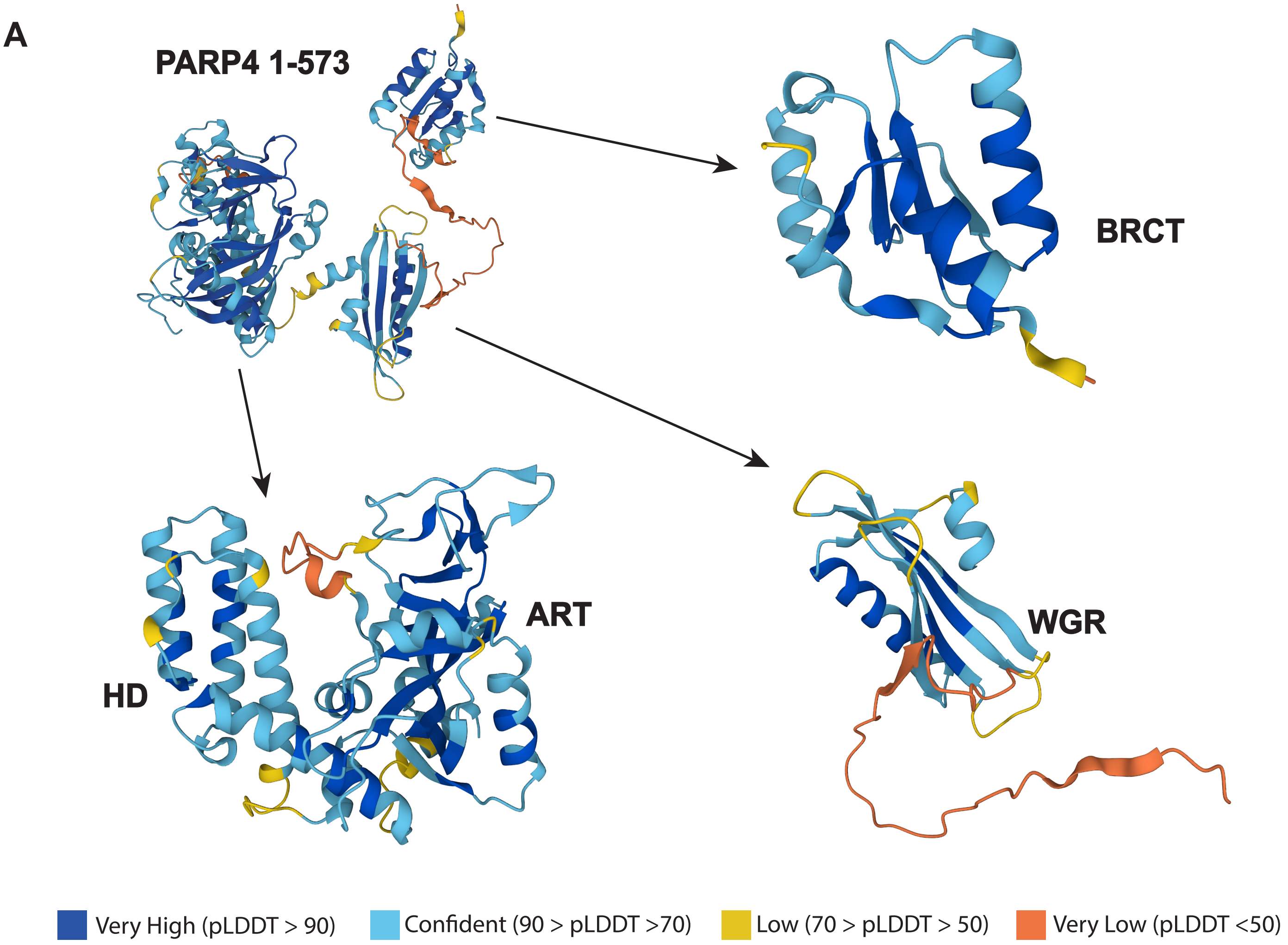
A
PARP4 1-573
BRCT
HD
ART
WGR
Very High (pLDDT > 90)
Confident (90 > pLDDT >70)
Low (70 > pLDDT > 50)
Very Low (pLDDT <50)

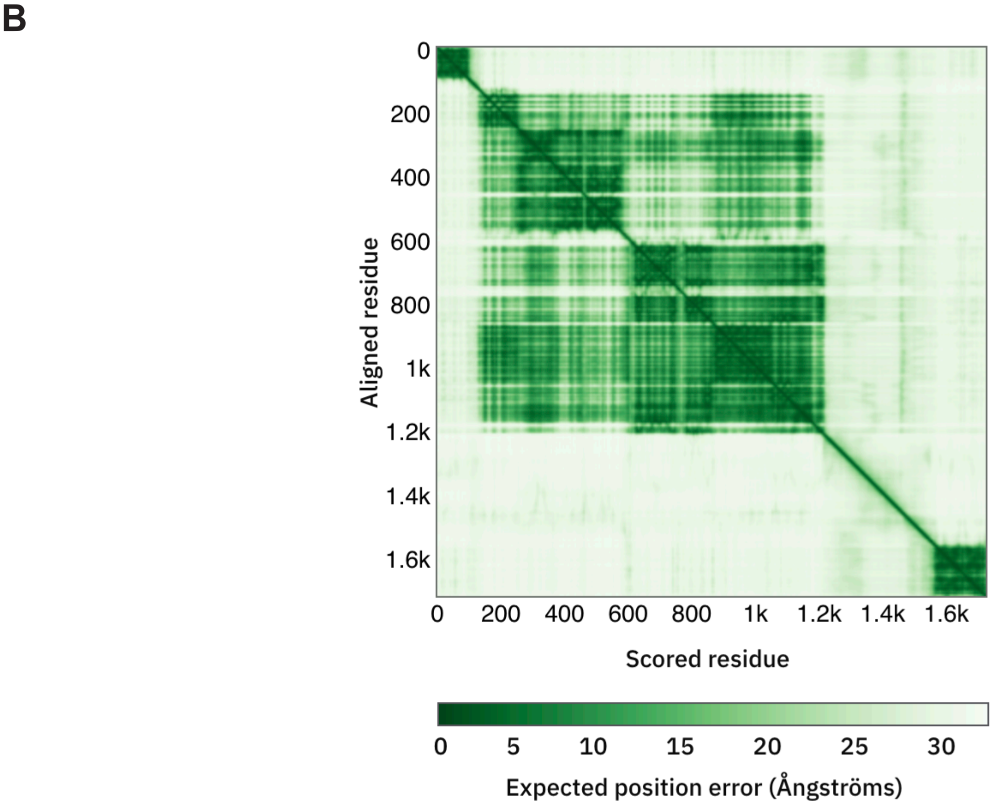
B
0
200
400
600
800
1k
1.2k
1.4k
1.6k
Aligned residue
Scored residue
0
5
10
15
20
25
30
Expected position error (Ångströms)

Figure S5. AlphaFold analysis of PARP4 structure

A. AlphaFold structure prediction of PARP4 N-terminal domains (residues 1-573). The structures are colored by the confidence scores for each residue of the predicted model (pLDDT).

B. Predicted aligned error (PAE) plot for the entire PARP4 sequence (residues 1-1724). The square clusters of darker green areas typically represent domains, with low coordinate position error values across long stretches of the sequence. Contacts between domains can be inferred from the medium green shaded areas that connect the darker green squares. For example, the dark green box in the top left represents the BRCT domain, and the dark green box in the bottom right represents the MVP-ID. These domains are not expected to have contacts with other domains, based on the high values of expected positional error (white coloring) between the residues of these domains and residues in other regions of the structure. In contrast, the WGR domain (~140 to 240) is predicted to have contacts with the catalytic domain (~240 to 570), since the expected position error between these two segments is relatively low (medium green coloring).

Figure S6.

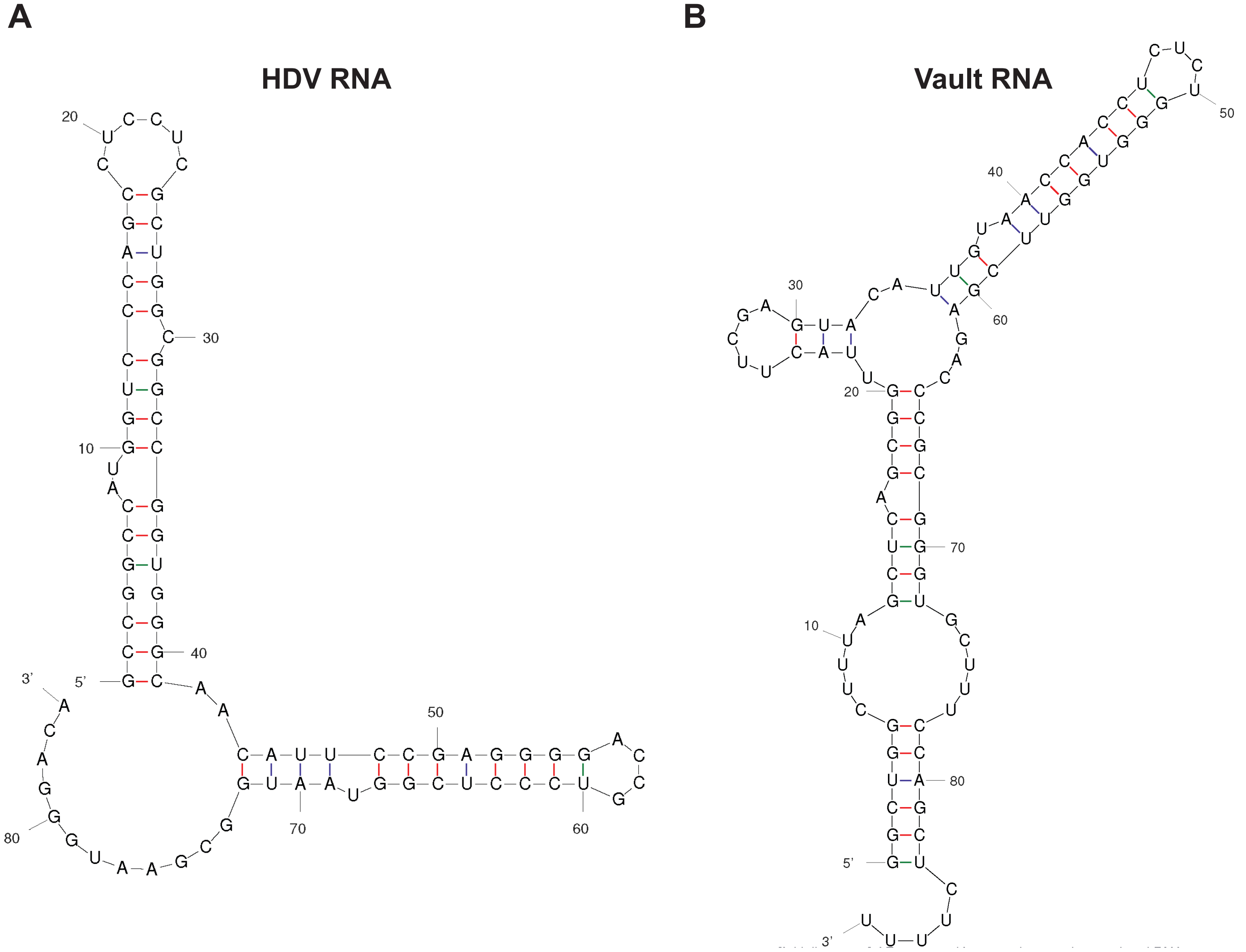
A
HDV RNA
5'
3'
10
20
30
40
50
60
70
80
B
Vault RNA
5'
3'
10
20
30
40
50
60
70
80

Figure S6. Secondary structures of vtRNA and HDV RNA

A. Secondary structure of the HDV RNA used in this study, as predicted by Mfold [42].

B. Secondary structure of the vtRNA used in this study, as predicted by Mfold [42].

Figure S7.

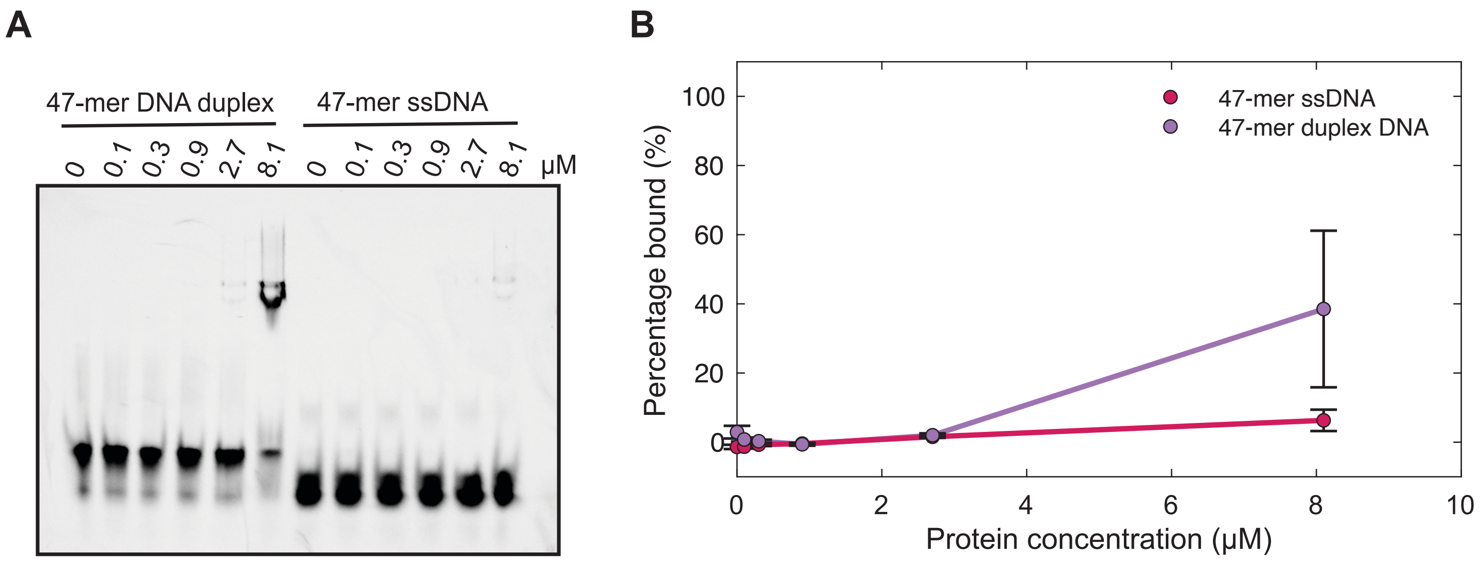
A
47-mer DNA duplex
47-mer ssDNA
0
0.1
0.3
0.9
2.7
8.1
0
0.1
0.3
0.9
2.7
8.1
μM
B
Percentage bound (%)
100
80
60
40
20
0
0
2
4
6
8
10
Protein concentration (μM)
47-mer ssDNA
47-mer duplex DNA

Figure S7. EMSA analysis of PARP4 interaction with DNA

A. EMSA using 47-base pair duplex DNA, or 47-nucleotide single-stranded DNA, both with fluorescent Cy3 labeling. BRCT-WGR-CAT was incubated with the DNA molecules at the indicated concentrations. This is a representative experiment that has been repeated two times.

B. Quantification of the two repeats of the EMSA shown in panel A. The data points represent the average and the error bars represent standard deviation of two independent experiments.
